# The total synthesis of K-252c (staurosporinone) *via* a sequential C–H functionalisation strategy†
†Electronic supplementary information (ESI) available: ^1^H and ^13^C NMR spectra, and crystallographic data. CCDC 1431476. For ESI and crystallographic data in CIF or other electronic format see DOI: 10.1039/c5sc04399a


**DOI:** 10.1039/c5sc04399a

**Published:** 2016-01-21

**Authors:** J. C. Fox, R. E. Gilligan, A. K. Pitts, H. R. Bennett, M. J. Gaunt

**Affiliations:** a Department of Chemistry , Department of Cambridge , Lensfield Road , Cambridge , CB2 1EW , UK . Email: mjg32@cam.ac.uk

## Abstract

A synthesis of the bioactive indolocarbazole alkaloid K-252c (staurosporinone) *via* a sequential C–H functionalisation strategy is reported. The route exploits direct functionalisation reactions around a simple arene core and comprises of two highly-selective copper-catalysed C–H arylations, a copper-catalysed C–H amination and a palladium-catalysed C–H carbonylation, which build up the structural complexity of the natural product framework.

## Abstract 2

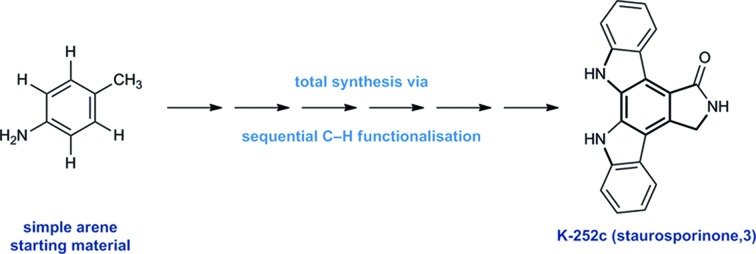
A synthesis of the bioactive indolocarbazole alkaloid K-252c (staurosporinone) *via* a sequential C–H functionalisation strategy is reported.

## 

The indolocarbazole alkaloids K-252a–d (**1–4**) are microbial metabolites first isolated in 1986 from culture broths of *Nocardiopsis* sp. K-252 and *Nocardiopsis* sp. K-290 (Fig. 1a).^1^ The compounds were found to be potent inhibitors of protein kinase C, an enzyme family known to play a critical role in a myriad of signal transduction pathways associated with metabolism, gene expression, membrane transport and cell proliferation.^2^ Consequently, the protein kinase C family of proteins has become an important therapeutic target for several disease classes, particularly in oncology, resulting in sustained interest in the design and development of inhibitors as potential pharmaceutical agents.^3^

**Fig. 1:**
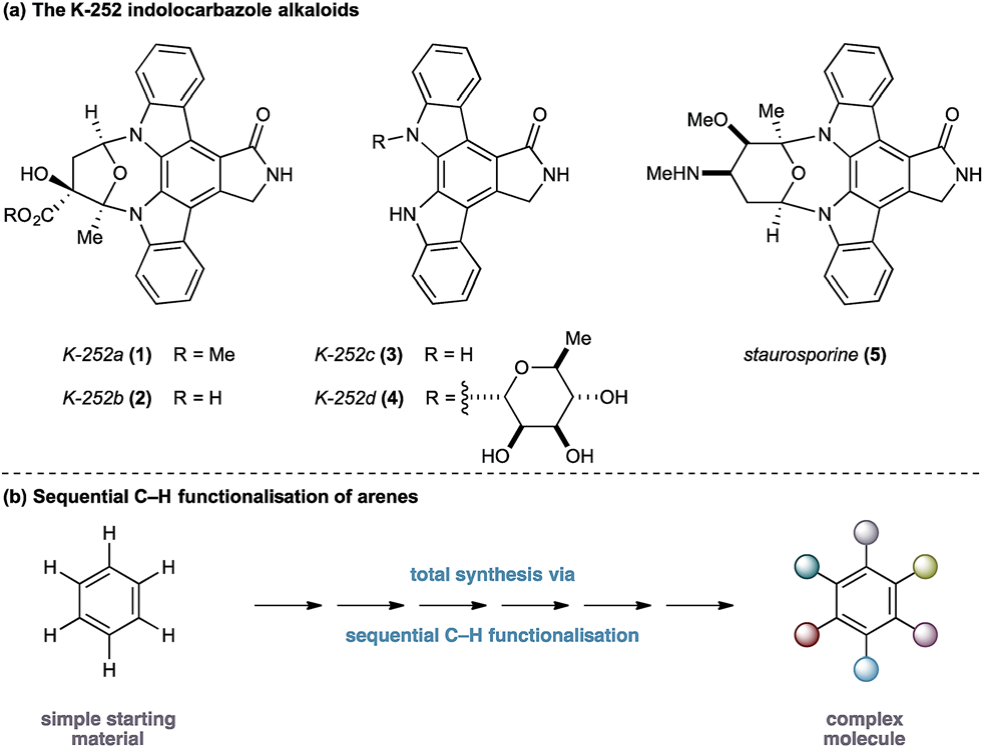
Strategy for the synthesis of K-252 indolocarbazole alkaloids.

In light of their intriguing biological properties and potential as pharmaceutical agents, the indolocarbazole alkaloids have attracted considerable interest from the synthetic community. In particular K-252c, (**3**, staurosporinone),^4^ the potential biosynthetic precursor and aglycone of staurosporine **5**,^5^ has attracted a number of elegant syntheses. We were attracted to these natural products because of their interesting biological activity and the unusual functional pattern of the hexasubstituted arene framework. Our group has a long standing interest in the synthesis of natural products that rely on strategies based on the sequential functionalisation of the C–H bonds in simple aromatic building blocks (Fig. 1b).^6^–^8^ We envisaged that K-252c, **3**, could provide a platform to further extend this total synthesis strategy by orchestrating a series of direct functionalisations on a readily available aniline to form the fully substituted benzene core of the target molecule. Furthermore, such a modular strategy would be amenable to the preparation of analogues of this important scaffold. Herein we report a concise synthesis of the indolocarbazole alkaloid K-252c starting from a commercial toluidine starting material. Seven direct functionalisations are used to transform C–H bonds of the aniline into the substituents required for the hexasubstituted arene core of the natural product architecture. Despite the increasingly complex architecture that results from each step, a notable feature of this synthesis is the exquisite selectivity of each C–H transformation, thereby highlighting the efficacy of sequential C–H functionalisation strategies.^9^

At the outset of our design stage, we questioned whether *p*-toluidine, a simple commercial aniline, could be used to launch a sequential C–H functionalisation strategy for the synthesis of K-252c, **3**, (Scheme 1). Many of the previous syntheses use a common strategy to assemble the indolocarbazole framework that is based on the union of two indole molecules with a synthetic precursor to the lactam ring system, followed by an oxidative electrocyclisation to form the poly(hetero)aromatic framework.^4^,^5^ Most elegant among these syntheses is the work of Wood and co workers who developed a concise approach to this class of natural products.5*c* Despite the simplicity of this type of strategic disconnection, it remains difficult to engineer an unsymmetrical indolocarbazole framework from such a convergent route. As part of our synthetic strategy towards staurosporinone, we envisaged that a sequential C–H functionalisation approach might benefit from late-stage carbazole formations in order to modulate the electron density of the central arene core (Fig. 2). The lactam would originate from a C–H carbonylation directed by the benzylamine motif, which itself could be introduced through a radical-mediated benzylic oxidation. These disconnections would reveal a teraryl framework **6**, displaying an amine motif that would control the selective installation of the adjacent nitro group and a series of *ortho*- and *meta*-C–H arylations, leading back to *p*-toluidine. Taken together, the amine motif of the aniline directly controls four out of seven direct functionalisations on the aromatic framework of this complex molecule.

**Scheme 1:**
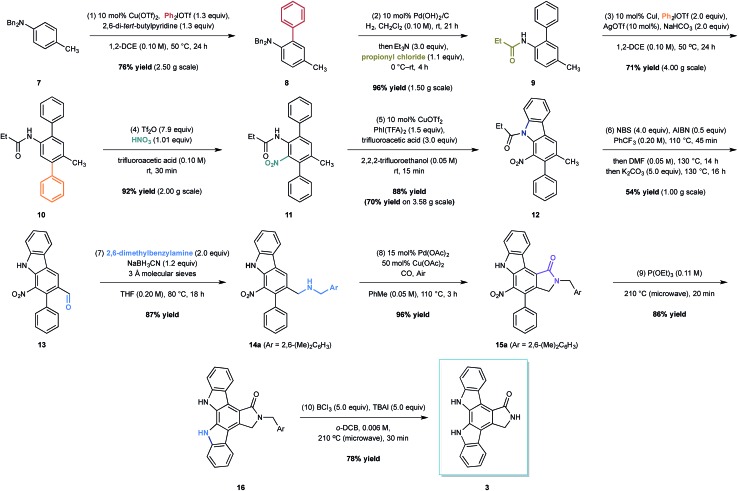
The synthesis of K-252c.

**Fig. 2:**
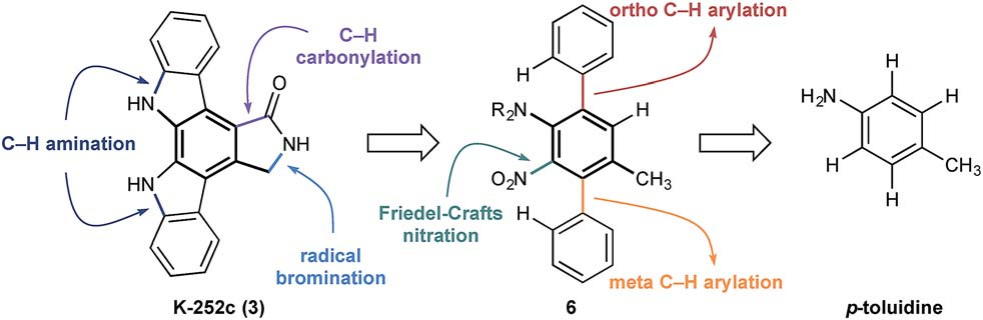
Retrosynthetic analysis of K-252c **3**.

The total synthesis of K-252c commenced from the readily available dibenzyl *p*-toluidine **7**, available in multigram quantities (Scheme 1). The first C–H functionalisation of the arene core involved the union of the commercial diphenyliodium triflate with **7** through the action of a simple copper(ii) catalyst, a biaryl coupling strategy recently developed by our group.^10^ The *ortho*-selective arylation proceeded in good yield and on a multigram scale to form **8**; we did not detect any of the corresponding di-*ortho*-phenylation product, which is often a problem with comparative metal-catalysed arylations. We believe that the selectivity of this process is due to the low reactivity of the product to subsequent arylations. A clash between the *ortho*-phenyl substituent and the *N*,*N*-dibenzyl group forces the amino group to rotate to alleviate the steric interaction; the lone pair on the nitrogen rotates out of conjugation with the arene, reducing the arene nucleophilicity and making it less reactive towards a second arylation.

To install the second aryl group, we envisaged the use of a copper-catalysed *meta*-arylation that has also been recently developed in our laboratory. To achieve this, however, the *N*-benzyl substituents needed to be switched to a carbonyl-containing group, which is important to impart the desired *meta*-selective arylation.^11^ The nature of the carbonyl group needed to carefully considered with respect to both the effectiveness in the *meta*-arylation, its stability to subsequent reaction conditions and its compatibility with the oxidative C–H amination that would be required to form the carbazole at a later point in the synthesis. We had previously shown that *meta*-arylation was best effected by a pivalamide group, but there are no examples of C–H aminations using amines derived with this bulky group. In contrast, *meta*-arylation directed by simple acetamides was often low yielding due to competitive amide cleavage, although there are a number of C–H aminations that can be initiated by this simple amide motif. In balancing these factors we elected to investigate the propionyl group in the hope that the extra size of the ethyl substituent would render the amide less liable to cleavage, but would still prove effective in the carbazole formation step. Therefore, a one-pot hydrogenolysis and subsequent carbamoylation with propionyl chloride enabled the formation of anilide **9** in excellent 96% yield on a multi-gram scale. Pleasingly, a slight modification to our copper-catalysed *meta*-arylation worked well and treatment of anilide **9** with diphenyliodonium triflate in the presence of copper(i) iodide, silver(i) triflate (providing an *in situ* source of copper(i) triflate) and solid sodium hydrogen carbonate (to buffer the reaction in light of the formation of triflic acid as a by-product) produced the teraryl intermediate **10** in 71% yield on a 4 g scale (the mass balance was unreacted starting material).

With teraryl **10** in hand, efforts next focused on the addition of the second nitrogen substituent. It was envisaged that installation of a nitro group, which could be subsequently employed in a late-stage reductive cyclisation to a carbazole, would be the optimal strategy. Treatment of **10** with concentrated nitric acid in a cooled solution of trifluoroacetic anhydride and trifluoroacetic acid yielded the desired product in 60% yield, although attempts to scale-up the reaction were unsuccessful, resulting in lower yields. Interestingly, we found that replacing the trifluoroacetic anhydride with trifluoromethanesulfonic anhydride, a modification of an underutilised aromatic nitration protocol developed by Hill and co-workers,^12^ led to significant improvements in both the yield and selectivity. Furthermore, the modified conditions were scalable, enabling an exquisitely selective and gram scale reaction to produce the nitro-arene **11** in 92% yield.

With both nitrogenous groups installed on the central arene framework, attention was turned to the C–H amination to form one of the two-carbazole units. Several metal catalysed C–H aminations to carbazoles have been developed in recent years,^13^ but unfortunately, we found that palladium-catalysed methods for carbazole formation were either capricious or resulted in none of the desired product when applied to our system.^14^ Pleasingly, the copper-catalysed oxidative C–H amination protocol developed by Chang and co-workers employing phenyliodide bis-trifluoroacetate and copper(ii) triflate effected the carbazole formation (to **12**) in good yield (70% on 3.58 g scale, 88% on 100 mg scale).^15^

We next turned to the installation of the γ-lactam motif that we speculated could arise from an advanced benzaldehyde. Despite the availability of a number of methods to affect to the oxidation of the benzylic methyl group to a benzaldehyde, all attempts to directly access **13** proved unsuccessful. This is possibly due to the deactivating effect of the resident nitro group. To overcome these difficulties, a selective radical bis-bromination of the benzylic position (to **12a**, Scheme 2),16*a* followed by treatment with DMF and K_2_CO_3_ afforded the desired aldehyde functionality with concomitant cleavage of the carbazole *N*-protecting group.16*b* This sequence could be combined into a one-pot procedure to supply the desired aldehyde in 54% yield on a gram scale.

**Scheme 2:**
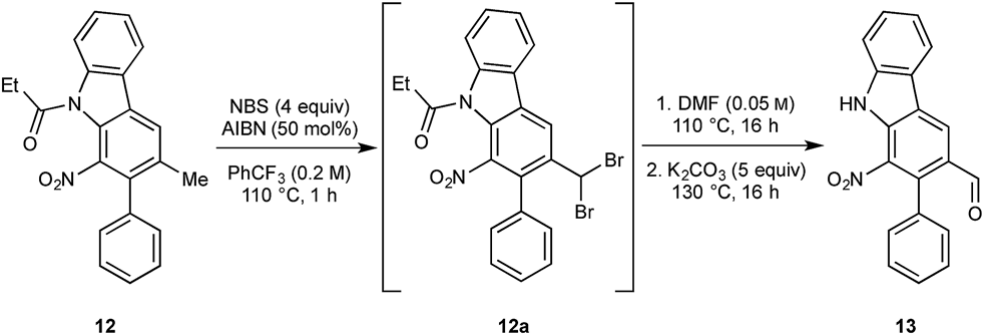
Telescoped synthesis of aldehyde **13**.

While we reasoned that reductive amination of aldehyde **13** with a suitable amine would lead to a precursor for the proposed C–H carbonylation to form the γ-lactam, we were conscious that primary amines coordinate strongly to metals such as palladium and often inhibit catalytic activity. Therefore, we elected to carry out the reductive amination with a benzylic amine with the intention of adopting Orito's C–H carbonylation to the desired protected γ-lactam.^17^ However, the choice of amine required further consideration as the use of benzyl amine would lead to selectivity problems in the carbopalladation step of the C–H carbonylation (selectivity between **I** and **II**, Scheme 3a). Mindful of this, we prepared a number of benzylamine variants displaying *ortho*-substituents to block the competitive C–H activation (Scheme 3b). We were pleased to find that 2,6-dimethylbenzylamine performed well in both the reductive amination and the palladium-catalysed C–H carbonylation to form γ-lactam **15a**.

**Scheme 3:**
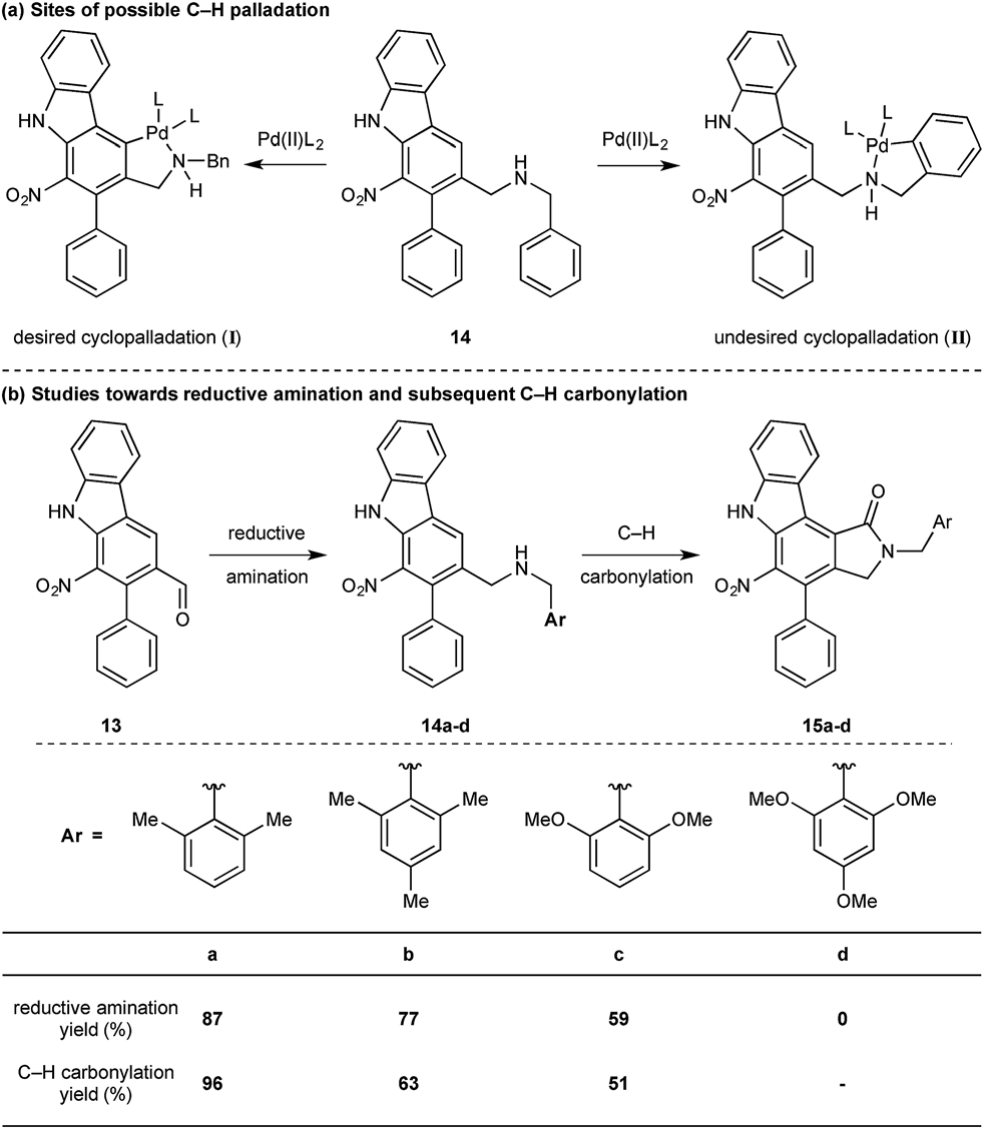
Optimisation of protecting group for C–H carbonylation.

With all but one ring now installed to the aromatic framework of staurosporinone, we turned our attention to the final C–H amination. After extensive experimentation we found that ring closure based on reductive metal-catalysed C–H aminations from the nitro group or oxidative C–H aminations from amine derivatives failed to produce any of the desired carbazole.^13^–^15^ However, we were delighted to find that a classical reductive Cadogan cyclisation from **15a**,^18^ utilised by Raphael *et al.* in their pioneering work on staurospoinone,4*c* produced the target carbazole **16** in modest yield. While these initial reactions, using P(OEt)_3_ to generate the requisite nitrene species, were low yielding (36% yield), we found that through careful monitoring of the reaction time and temperature (20 min at 210 °C), **16** could be isolated in 85% yield without the need for column chromatography. The final deprotection to afford K-252c **3** proved to be challenging, but after a survey of a range of standard *N*-debenzylation techniques we found that treatment of lactam **16** with TBAI and BCl_3_, conditions developed by Coe and co-workers for the deprotection of primary alkyl aryl ethers,^19^ afforded K-252c **3** in 78% yield, the spectral data for which were consistent with those reported in the literature.

## Conclusions

In summary, we have successfully applied a sequential C–H bond functionalisation strategy to the synthesis of K-252c. The overall yield if the synthesis from a readily available aniline (**7**) is 12.7%. The sequence of direct functionalisations follows a logical order of reactivity that sees the amine group of the starting toluidine control four of the C–H functionalisations. The synthesis showcases transformations comprising of two selective copper-catalysed C–H arylations, a highly selective electrophilic nitration, two C–H amination protocols to form carbazoles, a benzylic methyl oxidation and a palladium-catalysed C–H carbonylation. The synthesis is amenable to scale-up, providing access to intermediates on gram or multi-gram scale. Furthermore, it is envisaged that this modular approach could be used to provide rapid access to analogues of this biological important class of molecule, as well as complex hexasubstituted benzenes.^20^ Current research in our laboratory is focused on applying this sequential C–H functionalisation strategy to the synthesis of staurosporinone analogues and other complex natural products that could benefit from such a strategy.

## Supplementary Material

Supplementary information

Crystal structure data
