# Supplementary material for: The total synthesis of K-252c (staurosporinone) via a sequential C–H functionalisation strategy
Source: Chem Sci. 2016 Jan 21;7(4):2706–10. doi: 10.1039/c5sc04399a (PMC5477024; doi:10.1039/c5sc04399a)

Electronic Supplementary Material (ESI) for Chemical Science.
This journal is © The Royal Society of Chemistry 2016

The Total Synthesis of K-252c (Staurosporinone) *via* a Sequential C–H Functionalisation Strategy

J. C. Fox,[†] R. E. Gilligan,[†] A. K. Pitts, H. R. Bennett and M. J. Gaunt*

Department of Chemistry, University of Cambridge,
Lensfield Road, Cambridge CB2 1EW, United Kingdom.
*mjg32@cam.ac.uk

Supporting Information
Experimental procedures and data
^{1}H and ^{13}C NMR spectra

Contents

General Information 3

EXPERIMENTAL PROCEDURES 5

N,*N*-Dibenzyl-4-methylaniline (7) 6

N,*N*-Dibenzyl-5-methyl-[1,1'-biphenyl]-2-amine (8) 7

N-(5-Methyl-[1,1'-biphenyl]-2-yl)propionamide (9) 8

N-(5'-Methyl-[1,1':4',1''-terphenyl]-2'-yl)propionamide (10) 9

N-(5'-Methyl-3'-nitro-[1,1':4',1''-terphenyl]-2'-yl)propionamide (11) 10

1-(3-Methyl-1-nitro-2-phenyl-9*H*-carbazol-9-yl)propan-1-one (12) 11

1-(3-(Dibromomethyl)-1-nitro-2-phenyl-9*H*-carbazol-9-yl)propan-1-one (12a) 12

1-Nitro-2-phenyl-9*H*-carbazole-3-carbaldehyde (13) 13

N-(2,6-Dimethylbenzyl)-1-(1-nitro-2-phenyl-*9H*-carbazol-3-yl)methanamine (14a) 15

1-Mesityl-*N*-((1-nitro-2-phenyl-*9H*-carbazol-3-yl)methyl)methanamine (14b) 16

N-(2,6-Dimethoxybenzyl)-1-(1-nitro-2-phenyl-*9H*-carbazol-3-yl)methanamine (14c) 17

2-(2,6-Dimethylbenzyl)-5-nitro-4-phenyl-3,6-dihydropyrrolo[3,4-c]carbazol-1(*2H*)-one (15a) 18

5-Nitro-4-phenyl-2-(2,4,6-trimethylbenzyl)-3,6-dihydropyrrolo[3,4-c]carbazol-1(*2H*)-one (15b) 19

2-(2,6-Dimethoxybenzyl)-5-nitro-4-phenyl-3,6-dihydropyrrolo[3,4-c]carbazol-1(*2H*)-one (15c) 20

6-(2,6-Dimethylbenzyl)-6,7,12,13-tetrahydro-5*H*-indolo[2,3-a]pyrrolo[3,4-c]carbazol-5-one (16) 21

Staurosporinone (3) 22

NMR SPECTRA 24

N,*N*-Dibenzyl-4-methylaniline (7) 25

N,*N*-Dibenzyl-5-methyl-[1,1'-biphenyl]-2-amine (8) 26

N-(5-Methyl-[1,1'-biphenyl]-2-yl)acetamide *N*-(5-Methyl-[1,1'-biphenyl]-2-yl)propionamide (9) 27

N-(5'-Methyl-[1,1':4',1''-terphenyl]-2'-yl)propionamide (10) 28

N-(5'-Methyl-3'-nitro-[1,1':4',1''-terphenyl]-2'-yl)propionamide (11) 29

1-(3-Methyl-1-nitro-2-phenyl-9H-carbazol-9-yl)propan-1-one (12) 30

1-(3-(Dibromomethyl)-1-nitro-2-phenyl-9*H*-carbazol-9-yl)propan-1-one (12a) 31

1-Nitro-2-phenyl-9*H*-carbazole-3-carbaldehyde (13) 32

N-(2,6-Dimethylbenzyl)-1-(1-nitro-2-phenyl-9*H*-carbazol-3-yl)methanamine (14a) 33

1-Mesityl-*N*-((1-nitro-2-phenyl-*9H*-carbazol-3-yl)methyl)methanamine (14b) 34

N-(2,6-Dimethoxybenzyl)-1-(1-nitro-2-phenyl-*9H*-carbazol-3-yl)methanamine (14c) 35

2-(2,6-Dimethylbenzyl)-5-nitro-4-phenyl-3,6-dihydropyrrolo[3,4-c]carbazol-1(2H)-one (15a) 36

5-Nitro-4-phenyl-2-(2,4,6-trimethylbenzyl)-3,6-dihydropyrrolo[3,4-c]carbazol-1(*2H*)-one (15b) 37

2-(2,6-Dimethoxybenzyl)-5-nitro-4-phenyl-3,6-dihydropyrrolo[3,4-c]carbazol-1(*2H*)-one (15c) 38

6-(2,6-Dimethylbenzyl)-6,7,12,13-tetrahydro-5*H*-indolo[2,3-a]pyrrolo[3,4-c]carbazol-5-one (16) 39

Staurosporinone (3) 40

General Information

Solvents: All anhydrous solvents were dried by standard techniques and freshly distilled before use. Diethyl ether and tetrahydrofuran were distilled from calcium hydride and lithium aluminium hydride; acetonitrile, dichloromethane and toluene from calcium hydride; and triethylamine from potassium hydroxide.

Reagents: All reagents were purified by standard procedures[1] or used as obtained from commercial sources. Copper(II) triflate was purchased from Alfa Aesar, dried under vacuum at 80 °C and stored under nitrogen before use. 4-Bromoindole was purchased from Apollo Scientific and used as supplied. Di-*tert*-butylpyridine was purchased from Molecular and used as supplied.

Chromatography: All flash chromatography was carried out using dry packed Merck 9385 Kieselgel 60 silica gel and thin layer chromatography was carried out on Merck Kieselgel 60 PF254 0.2 mm plates. Visualisation was accomplished using ultra violet light (254 nm) and chemical staining with ceric ammonium molibdate or basic potassium permanganate solutions as appropriate.

Equipment: Liquid Chromatography-Mass Spectrometry (LCMS) was performed on a Shimadzu UFLC-XR/LCMS 2020 system using a Shim-pack XR-ODS column (C18, 2.2μm, 3.0 mm x 50 mm). The LCMS acetonitrile (Rathburn) was pre-treated with water (5% vol) and formic acid (12.5 mM) and the LCMS water (Rathburn) was pre-treated with formic acid (25 mM) and ammonium acetate (10 mM). Gas Chromatography-Mass Spectrometry (GCMS) was performed on a Shimadzu MDGC/GCMS-2010 system. Microwave experiments were performed in a Biotage® Initiator+ System equipped with Robot Eight.

Reactions: All reactions were carried out using oven dried glassware and under an atmosphere of nitrogen unless otherwise stated.

Data Collection: Corrected ^{1}H NMR spectra were recorded on a Bruker DPX 400 or 500 spectrometer and quoted to 0.01 ppm in deuterochloroform ($CDCl_3$), unless stated otherwise. ^{13}C NMR spectra were recorded at 100 or 125 MHz and quoted to 0.1 ppm on the same machines. Chemical shifts (δ) are quoted in parts per million (ppm) relative to residual solvent ($CDCl_3$: δ = 7.26 ppm for ^{1}H NMR and δ = 77.0 for ^{13}C NMR, acetone-d_6: δ = 2.05 ppm for ^{1}H NMR and δ = 29.8, 206.3 for ^{13}C NMR, DMSO-d_6: δ = 2.05 ppm for ^{1}H NMR and δ = 39.55 for ^{13}C NMR, acetonitrile-d_3: δ = 1.94 ppm for ^{1}H NMR and δ = 1.3, 118.3 for ^{13}C NMR); Coupling constants (*J*) are corrected and quoted in Hz to the nearest 0.1 Hz. The following abbreviations are used to indicate the multiplicity of the

[1] Armarego, W. L. F.; Perrin, D. D., *Purification of Laboratory Chemicals*, 5th Ed., Butterworth-Heinemann, **1996**.

signals: s = singlet; d = doublet; t = triplet; q = quartet; qn = quintet; st = sextet, sp = septet; m = multiplet; br. = broad; app. = apparent; and associated combinations, e.g. dd = doublet of doublets. The temperature of the acquisition of the NMR spectra was 298 ± 3K. DEPT135, nOe experiments and 2-dimensional experiments (COSY, HMBC and HMQC) were used to support assignments where appropriate but are not included. High resolution mass spectra (HRMS) were measured on a Micromass Q-TOF spectrometer using EI (electron impact) or ES (electrospray ionisation) techniques at the Department of Chemistry, University of Cambridge or at the EPSRC Mass Spectrometry Service at the University of Swansea. Infrared (IR) spectra were recorded on a Perkin Elmer 1FT-IR Spectrometer fitted with an ATR sampling accessory as either solids or neat films, either through direct application or deposited in $CHCl_3$, with absorptions reported in wavenumbers (cm^{-1}). Melting points (m.p.) were recorded using a Galenkamp hot stage apparatus. X-ray crystallography was performed on a Nonius Kappa CCD at the Cambridge University Chemistry X-Ray by Dr John E. Davies and data was deposited with the Cambridge Crystallographic Database.

EXPERIMENTAL PROCEDURES

***N*,*N*-Dibenzyl-4-methylaniline (7)**

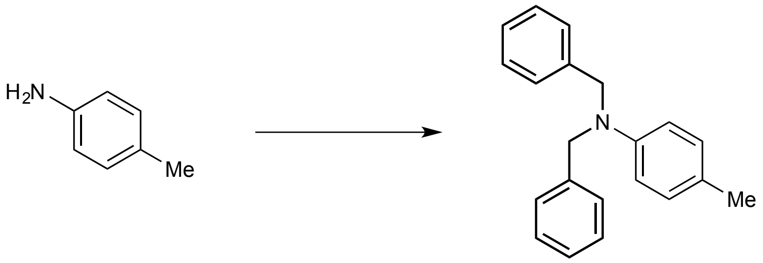

Benzyl bromide (41.63 mL, 350 mmol, 3.5 equiv) was added to a suspension of *p-toluidine* (10.72 g, 100 mmol, 1.0 equiv) and potassium carbonate (41.46 g, 300 mmol, 3.0 equiv) in *N*,*N*-dimethylformamide (150 mL). The reaction mixture was heated at 50 °C for 1 hours after which time the mixture was cooled, diluted with ethyl acetate and washed with water and saturated aqueous NaCl solution. The organics were dried over magnesium sulphate and concentrated *in vacuo*. Purification by flash column chromatography (0–5% dichloromethane in 40–60 petroleum ether) yielded *N*,*N*-dibenzyl-4-methylaniline as a white solid (14.51 g, 48.15 mmol, 96%). Data in agreement with literature.[2]

^{1}H NMR (400 MHz, $CDCl_3$) δ 7.39–7.32 (m, 4H), 7.32–7.24 (m, 6H), 7.02 (d, *J* = 8.4 Hz, 2H), 6.71 (d, *J* = 8.8 Hz, 2H), 4.66 (s, 4H), 2.27 (s, 3H).

^{13}C NMR (100 MHz, $CDCl_3$) δ 147.2, 139.0 (2C), 129.9 (2C), 128.7 (4C), 126.9 (2C), 126.9 (4C), 126.0, 112.8 (2C), 54.6 (2C), 20.4.

[NMR spectra] [table of contents]

2. Watanabe, Y.; Tsuji, Y.; Ige, H.; Ohsugi, Y.; Ohta, T., *J. Org. Chem.* 1984, **49**, 3359–3363

_N,N_-Dibenzyl-5-methyl-[1,1'-biphenyl]-2-amine (8)

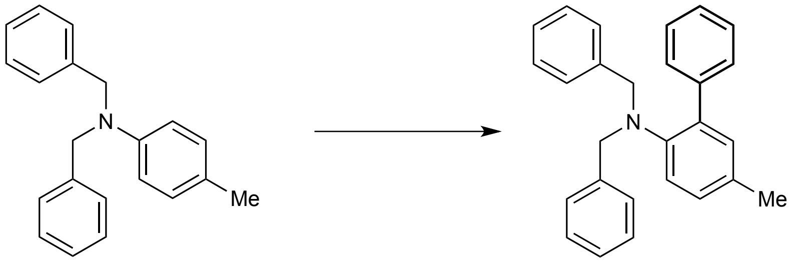

N,*N*-Dibenzyl-4-methylaniline (2.50 g, 8.70 mmol, 1.0 equiv), diphenyliodonium triflate (4.85 g, 11.27 mmol, 1.3 equiv), and copper(II) triflate (315 mg, 0.87 mmol, 10 mol%) were suspended in 1,2-dichloroethane (43.50 mL). 2,6-Di-*tert*-butylpyridine (2.45 mL, 11.31 mmol, 1.3 equiv) was then added *via* syringe, the flask was sealed, and the reaction stirred at 50 °C for 13 hours. The reaction mixture was allowed to cool to room temperature, washed with sodium bicarbonate and back-extracted with dichloromethane. The organic fractions were combined, dried over magnesium sulphate and concentrated *in vacuo*. Purification by flash column chromatography (5% dichloromethane in 40–60 petroleum ether) afforded *N*,*N*-dibenzyl-5-methyl-[1,1'-biphenyl]-2-amine (2.40 g, 6.61 mmol, 76%) as a crystalline white solid. Data in agreement with literature.[3]

^{1}H NMR (400 MHz, $CDCl_3$) δ: 7.49 (d, *J* = 7.6 Hz, 2H), 7.35 (t, *J* = 7.6 Hz, 2H), 7.27–7.21 (m, 1H), 7.17–7.06 (m, 6H), 7.00–6.90 (m, 6H), 6.82 (d, *J* = 8.0 Hz, 1H), 3.78 (s, 4H), 2.22 (s, 3H).
^{13}C NMR (100 MHz, $CDCl_3$) δ: 146.5, 141.8, 138.4 (2C), 136.8, 132.4, 132.3, 129.4 (2C), 129.1 (4C), 128.5, 128.3 (2C), 128.1 (4C), 126.9 (2C), 126.8, 121.8, 56.3 (2C), 20.9.

[NMR spectra] [table of contents]

3. Ciana, C.-L.; Phipps, R. J.; Brandt, J. R.; Meyer, F.-M.; Gaunt, M. J. *Angew. Chem. Int. Ed.* **2011**, *50*, 458

_N_-(5-Methyl-[1,1'-biphenyl]-2-yl)propionamide (9)

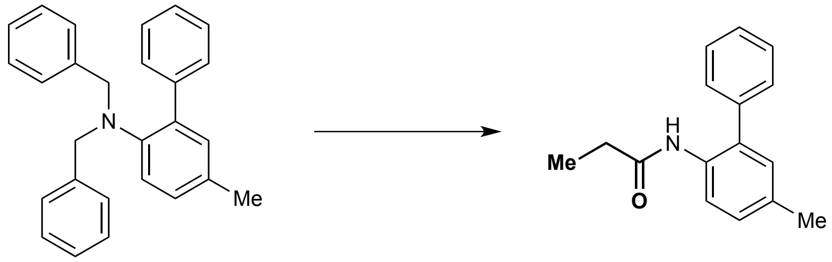

_N_,_N_-Dibenzyl-5-methyl-[1,1'-biphenyl]-2-amine (1.50 g, 4.13 mmol, 1.0 equiv) and palladium hydroxide on carbon (116 mg, 10 wt% Pd, 10 mol%) were combined and the reaction flask was evacuated and refilled with hydrogen three times. Dichloromethane (41.0 mL) was added and the reaction stirred at room temperature for 12 hours. The reaction mixture was cooled to 0 °C, triethylamine (0.69 mL, 4.97 mmol, 1.2 equiv) was added in one portion followed by dropwise addition of propionyl chloride (0.43 mL, 4.96 mmol, 1.2 equiv) over 20 min. The reaction mixture was allowed to warm to room temperature and stirred for 30 min, then filtered through a pad of Celite, dried over magnesium sulphate, and filtered through silica (25% dichloromethane in 40–60 petroleum ether) to afford _N_-(5-methyl-[1,1'-biphenyl]-2-yl)propionamide as a white solid (944 mg, 3.94 mmol, 96%).

m.p.: 109–111 °C.
R_f: 0.41 (20% dichloromethane in 40–60 petroleum ether).
IR ν_{max}/cm^{-1} (film): 3226, 3025, 2970, 1652, 1527, 1370, 1270, 1243, 1070, 820, 762, 696.
^{1}H NMR (400 MHz, $CDCl_3$) δ: 8.14 (d, _J_ = 8.0 Hz, 1H), 7.47 (app. t, _J_ = 7.2 Hz, 2H), 7.43–7.32 (m, 3H), 7.17 (d, _J_ = 8.0 Hz, 1H), 7.09 (br. s, 1H), 7.06 (br. s, 1H), 2.35 (s, 3H), 2.(q, _J_ = 7.6 Hz, 2H), 1.10 (t, _J_ = 7.6 Hz, 3H).
^{13}C NMR (100 MHz, $CDCl_3$) δ: 171.8, 138.5, 133.9, 132.4, 132.3, 130.6, 129.3 (2C), 129.1 (2C), 129.0, 127.9, 121.8, 30.8, 20.9, 9.7.
HRMS (n-ESI) (m/z): found 240.1384 $[M+H]^+$ ($[C_{16}H_{17}NO+H]^+$ requires 240.1383).

[NMR spectra] [table of contents]

N-(5'-Methyl-[1,1':4',1''-terphenyl]-2'-yl)propionamide (10)

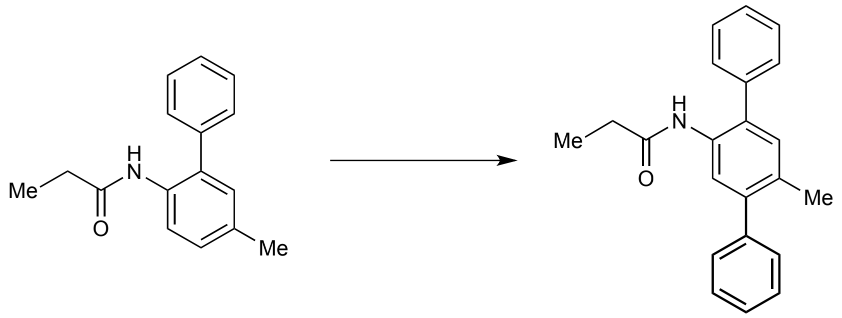

To a stirred mixture of *N*-(5-methyl-[1,1'-biphenyl]-2-yl)propionamide (4.00 g, 16.71 mmol, 1.0 equiv) in 1,2-dichloroethane (167 mL) at 50 °C was added diphenyliodonium triflate (9.34 g, 21.72 mmol), sodium hydrogen carbonate (2.80 g, 33.42 mmol.0), silver(I) triflate (430 mg, 1.68 mmol, 10 mol%) and copper(I) iodide (318 mg, 1.68 mmol, 10 mol%) in two equal portions over 30 min. The reaction mixture was then stirred for an additional 1 hour, allowed to cool to room temperature and diluted with dichloromethane. The organic layer was washed with 0.5 M HCl and saturated aqueous NaCl solution, dried over magnesium sulphate, filtered and concentrated onto silica *in vacuo*. Purification by flash column chromatography (0–2% diethyl ether in dichloromethane) yielded *N*-(5'-methyl-[1,1':4',1''-terphenyl]-2'-yl)propionamide as a white solid (3.75 g, 11.90 mmol, 71%).

m.p.: 148–150°C.
R_f: 0.35 (15% dichloromethane in 40–60 petroleum ether).
IR ν_{max}/cm^{-1} (film): 3191, 3023, 1641, 1581, 1532, 1482, 1390, 1232, 1075, 890, 766, 702.
^{1}H NMR (400 MHz, $CDCl_3$) δ 8.20 (s, 1H), 7.54–7.46 (m, 2H), 7.46–7.38 (m, 7H), 7.38–7.30 (m, 1H), 7.17 (s, 1H), 7.13 (br. s, 1H), 2.29 (s, 3H), 2.23 (q, *J* = 7.6 Hz, 2H), 1.11 (t, *J* = 7.6 Hz, 3H).
^{13}C NMR (100 MHz, DMSO-d_6) δ 172.4, 140.6, 140.5, 138.7, 135.7, 132.6, 132.4, 131.0, 128.9 (2C), 128.8 (2C), 128.3 (2C), 128.3 (2C), 127.2, 127.1, 28.8, 19.7, 9.7 (one carbon obscured by solvent).
HRMS (n-ESI) (m/z): found 316.1700 $[M+H]^+$ ($[C_{22}H_{21}NO+H]^+$ requires 316.1696).

[NMR spectra] [table of contents]

N-(5'-Methyl-3'-nitro-[1,1':4',1''-terphenyl]-2'-yl)propionamide (11)

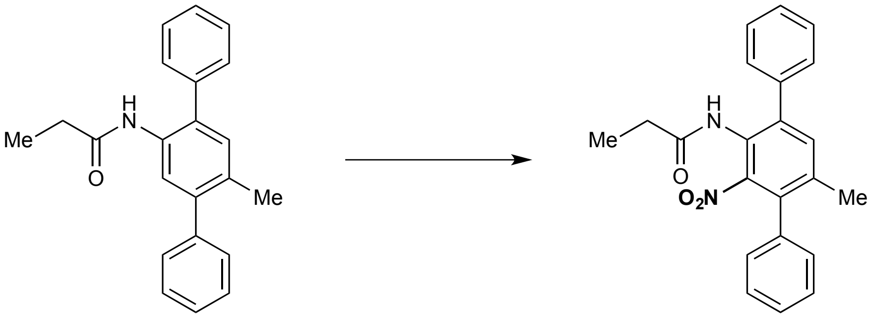

Original Conditions

To a solution of N-(5'-methyl-[1,1':4',1''-terphenyl]-2'-yl)propionamide (0.175 g, 0.555 mmol) in trifluoroacetic acid (2.81 mL) and trifluoroacetic anhydride (0.78 mL, 5.55 mmol) at 0 °C was added a solution of nitric acid (0.03 ml, 0.67 mmol) in trifluoroacetic acid (2.63 mL) slowly over 45 minutes. The reaction mixture was then stirred at 0 °C for a further 4 hours. Ice was added to the reaction mixture and stirred for 1 hour. The reaction mixture was extracted with ethyl acetate, washed with saturated sodium bicarbonate solution, dried over magnesium sulphate and concentrated in vacuo. Column chromatography in 10–30% diethyl ether in petroleum ether yielded N-(5'-methyl-3'-nitro-[1,1':4',1''-terphenyl]-2'-yl)propionamide **(11)** as a white solid (120 mg, 60%).

Improved Conditions

N-(5'-Methyl-[1,1':4',1''-terphenyl]-2'-yl)propionamide (2.0 g, 6.34 mmol, 1.0 equiv) was suspended in trifluoroacetic acid (31.0 mL) and trifluoromethanesulphonic anhydride (8.43 mL, 50.11 mmol, 7.9 equiv) before cooling to 0 °C. A solution of 70% nitric acid (411 µL, 6.39 mmol, 1.0 equiv) in trifluoroacetic acid (31.0 mL) was prepared and added to the reaction mixture over 30 min *via* syringe pump. The reaction mixture was immediately quenched by pouring onto ice-cold saturated sodium bicarbonate solution (150 mL) and neutralised with solid sodium bicarbonate with vigorous stirring. The aqueous layer was extracted with dichloromethane and the combined organics dried over magnesium sulphate, filtered and concentrated on silica *in vacuo*. Flash column chromatography (30–80% ethyl acetate in hexanes) provided N-(5'-methyl-3'-nitro-[1,1':4',1''-terphenyl]-2'-yl)propionamide (2.10 g, 5.82 mmol, 92%) as an off-white solid. Crystal structure deposited within the Cambridge Crystallographic Data Centre CCDC 1431476 (this contains the supplementary crystallographic data for compound **11**, and can be accessed free of charge *via* https://summary.ccdc.cam.ac.uk/structure-summary-form).

m.p.: 166–168 °C.

R_f: 0.35 (30% ethyl acetate in 40–60 petroleum ether).

IR ν_{max})/cm^{-1} (film): 3191, 3023, 1640, 1531, 1482, 1390, 1232, 1076, 890, 766, 702.

^{1}H NMR (400 MHz, DMSO-d_6) δ: 9.63 (s, 1H), 7.58 (s, 1H), 7.54–7.38 (m, 7H), 7.28 (dd, J = 6.4, 1.6 Hz, 2H), 2.14 (s, 3H), 2.00 (q, J = 8.0 Hz, 2H), 0.80 (t, J = 8.0 Hz, 3H).

^{13}C NMR (100 MHz, DMSO-d_6) δ: 173.1, 150.5, 141.1, 137.4, 137.2, 134.3, 133.1, 134.5, 128.8 (2C), 128.7 (2C), 128.6 (2C), 128.5, 128.3 (2C), 127.9, 124.4, 28.3, 19.9, 9.6.

HRMS (n-ESI) (m/z) found 361.1551 $[M+H]^+$ ($[C_{22}H_{20}N_2O_3+H]^+$ requires 361.1547).

[NMR spectra] [table of contents]

1-(3-Methyl-1-nitro-2-phenyl-9*H*-carbazol-9-yl)propan-1-one (12)

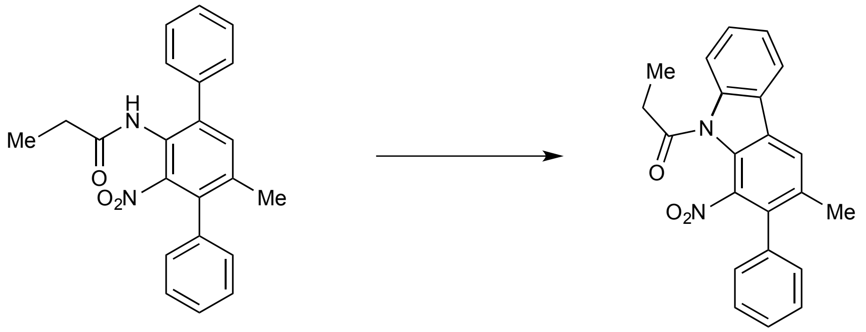

A solution of [bis(trifluoroacetoxy)iodo]benzene (3.58 g, 8.32 mmol, 1.5 equiv) in 2,2,2-trifluoroethanol (55.0 mL) was added *via* cannula over 20 min to a solution of *N*-(5'-methyl-3'-nitro-[1,1':4',1''-terphenyl]-2'-yl)propionamide (2.00 g, 5.55 mmol, 1.0 equiv), copper(II) triflate (46 mg, 0.13 mmol, 2.5 mol%) and trifluoroacetic acid (1.28 mL, 7.87 mmol, 1.4 equiv) in 2,2,2-trifluoroethanol (55.0 mL). The reaction was stirred at room temperature for 1 hour, diluted with dichloromethane, concentrated onto silica *in vacuo* and purified by flash column chromatography (25–75% dichloromethane in hexane) yielding 1-(3-methyl-1-nitro-2-phenyl-9H-carbazol-9-yl)propan-1-one (1.40 g, 3.89 mmol, 70%) as a yellow solid.

The product could be isolated as a yellow solid (44 mg, 0.122 mmol, 88%) if performed on a 0.14 mmol scale.

m.p.: 177–179 °C.

$\mathbf{R_f}$: 0.23 (50% dichloromethane in 40–60 petroleum ether).

IR ν_{max}/cm^{-1} (film): 2987, 2942, 2920, 1714, 1537, 1434, 1374, 1356, 1337, 1248, 1192, 1118, 1066, 900, 879, 783, 764, 754, 701.

^{1}H NMR (500 MHz, DMSO-d_6) δ: 8.43 (app. d, J = 0.5 Hz, 1H), 8.29 (dd, J = 7.8, 1.0 Hz 1H), 8.01 (d, J = 8.5 Hz, 1H), 7.63 (ddd, J = 8.5, 7.5, 1.5 Hz, 1H), 7.50–7.43 (m, 4H), 7.23 (dt, J = 6.5, 2.0 Hz, 2H,), 3.23 (q, J = 7.0 Hz, 2H), 2.15 (app. d, J = 0.5 Hz, 3H), 1.19 (t, J = 7.0 Hz, 3H).

^{13}C NMR (125 MHz, $CDCl_3$) δ: 173.9, 140.4, 139.1, 136.6, 134.4, 133.9, 128.9 (2C), 128.6 (2C), 128.4, 128.3, 128.2, 128.2, 125.5, 123.9, 123.0, 120.6, 114.8, 32.3, 21.4, 9.1.

HRMS (n-ESI) (m/z): found 376.1660 $[M+NH_4]^+$ ($[C_{22}H_{18}N_2O_3+NH_4]^+$ requires 376.1656).

[NMR spectra] [table of contents]

1-(3-(Dibromomethyl)-1-nitro-2-phenyl-9*H*-carbazol-9-yl)propan-1-one (12a)

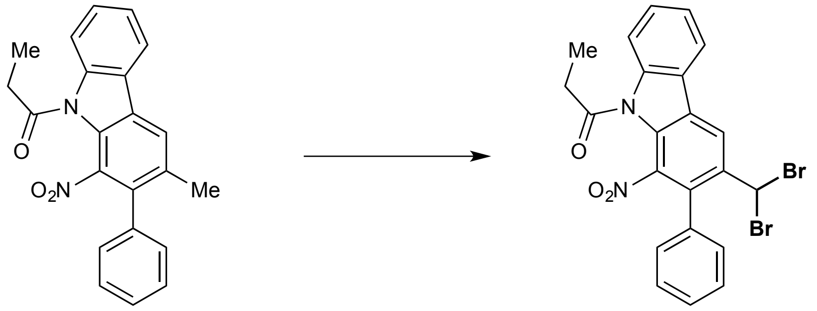

1-(3-Methyl-1-nitro-2-phenyl-9H-carbazol-9-yl)propan-1-one (250 mg, 0.70 mmol, 1.0 equiv), AIBN (57 mg, 0.35 mmol, 0.5 equiv), and NBS (496 mg, 2.79 mmol, 4.0 equiv) were suspended in α,α,α-trifluorotoluene (5 mL) and heated at 110 °C for 45 mins. The crude reaction mixture was concentrated onto silica *in vacuo* and purified by flash column chromatography (50% dichloromethane in 40–60 petroleum ether) affording 1-(3-(dibromomethyl)-1-nitro-2-phenyl-9*H*-carbazol-9-yl)propan-1-one (262 mg, 0.51 mmol, 73%) as a yellow solid.

m.p. : 222 °C (decomposition).
R_f: 0.34 (50% dichloromethane in 40–60 petroleum ether).
IR ν_{max}/cm^{-1} (film): 2987, 2919, 1720, 1541, 1467, 1419, 1371, 1344, 1247, 1198, 1147, 900, 748, 701.
^{1}H NMR (500 MHz, $CDCl_3$) δ: 8.85 (s, 1H), 8.22 (dq, *J* = 7.5, 0.5 Hz, 1H), 7.81 (d, *J* = 8.5 Hz, 1H), 7.64–7.60 (m, 1H), 7.54–7.47 (m, 4H), 7.37–7.32 (m, 2H) 6.35 (s, 1H), 3.13 (q, *J* = 7.5 Hz, 2H), 1.39 (t, *J* = 7.5 Hz, 3H).
^{13}C NMR (100 MHz, $CDCl_3$) δ: 174.1, 139.3, 138.7, 137.3, 133.3, 130.1, 130.0, 129.9, 129.4, 129.2, 129.0 (2C), 128.9 (2C), 124.8, 124.4, 123.7, 121.3, 114.6, 37.9, 32.6, 9.1.
HRMS (APCI) (m/z): found 533.9840 $[M+NH_4]^+$ ($[C_{22}H_{16}Br_2N_2O_3 + NH_4]^+$ requires 533.9845).

[NMR spectra] [table of contents]

1-Nitro-2-phenyl-9*H*-carbazole-3-carbaldehyde (13)

*From 1-(3-(dibromomethyl)-1-nitro-2-phenyl-9H-carbazol-9-yl)propan-1-one (**12a**)*;

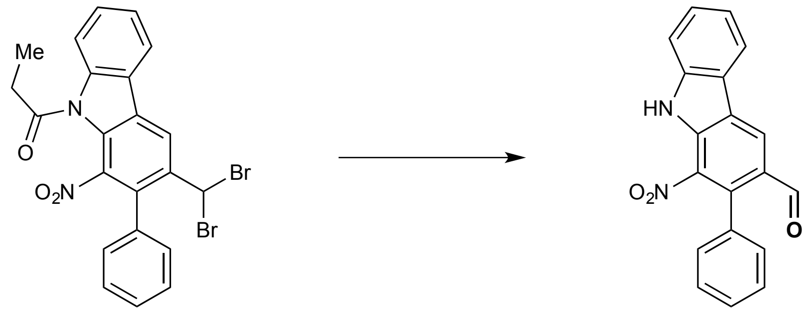

1-(3-(Dibromomethyl)-1-nitro-2-phenyl-9*H*-carbazol-9-yl)propan-1-one (200 mg, 0.39 mmol, 1.0 equiv) was suspended in α,α,α-trifluorotoluene (4.0 mL) and DMF (6.0 mL) and heated to 110 °C for 14 hours. Incomplete conversion was observed, and so the reaction mixture was heated to 120 °C for a further 11 hours. Potassium bicarbonate (261 mg, 1.89 mmol, 4.8 equiv) was added and the reaction mixture was heated to 120 °C for 12 hours. The reaction mixture was cooled and diluted with ethyl acetate, washed twice with deionised water, saturated aqueous NaCl solution, and separated. The organics were filtered though a pad of silica (eluting with dichloromethane), dried with magnesium sulphate, filtered and concentrated *in vacuo* to afford 1-nitro-2-phenyl-9*H*-carbazole-3-carbaldehyde (110 mg, 0.30 mmol, 76%) as a dark orange solid.

*From 1-(3-methyl-1-nitro-2-phenyl-9H-carbazol-9-yl)propan-1-one (**12**)*;

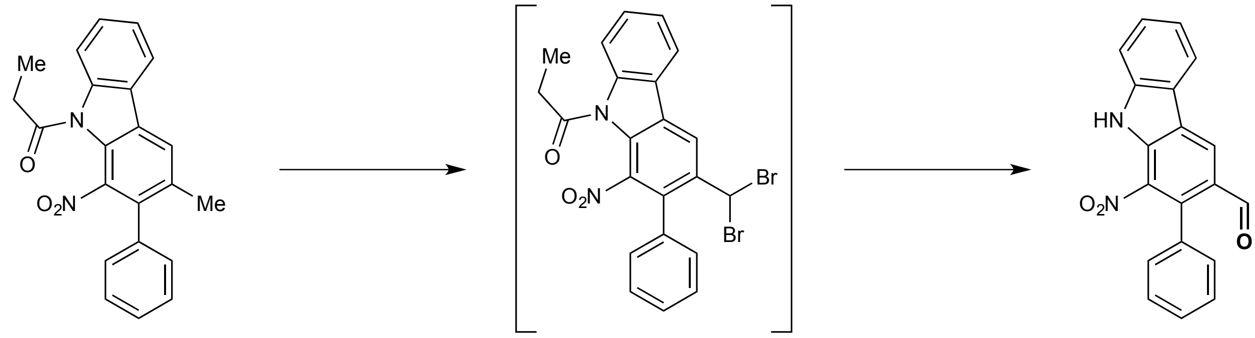

1-(3-Methyl-1-nitro-2-phenyl-9H-carbazol-9-yl)propan-1-one (1.00 g, 2.79 mmol, 1.0 equiv), AIBN (229 mg, 1.40 mmol, 0.5 equiv), and NBS (1.99 g, 11.16 mmol, 4.0 equiv) were suspended in α,α,α-trifluorotoluene (28.0 mL) and heated at 110 °C for 45 minutes. DMF (14.0 mL) was then added *via* syringe and the reaction mixture was heated at 130 °C for 14 hours, after which time potassium carbonate (1.93 g, 13.95 mmol, 5.0 equiv) was added and the reaction mixture was heated for a further 16 hours. The reaction mixture was cooled to room temperature, diluted with ethyl acetate, washed twice with water and saturated aqueous NaCl solution, and dried over magnesium sulphate before filtering and concentrating onto silica *in vacuo*. Flash column chromatography (10–70% dichloromethane in 40–60 petroleum ether) afforded 1-nitro-2-phenyl-9*H*-carbazole-3-carbaldehyde (480 mg, 1.52 mmol, 54%; 82% average yield per manipulation) as an orange solid.

m.p.: 280 °C (decomposition).

R_f: 0.25 (50% dichloromethane in 40–60 petroleum ether)

IR ν_{max}/cm^{-1} (film): 3348, 3958, 2920, 2888, 2852, 1681, 1634, 1599, 1513, 1497, 1425, 1367, 1337, 1322, 1289, 1254, 1213, 1203, 1173, 1143, 1011, 955, 885, 787, 754, 694.

^{1}H NMR (400 MHz, DMSO-d_6) δ: 12.40 (br. s, 1H), 9.56 (s, 1H), 9.04 (s, 1H), 8.45 (d, *J* = 7.6 Hz, 1H), 7.74 (d, *J* = 8.0 Hz, 1H), 7.60 (t, *J* = 7.6 Hz, 1H), 7.55–7.45 (m, 5H), 7.39 (t, *J* = 7.2 Hz, 1H).

^{13}C NMR (125 MHz, $CDCl_3$) δ: 189.8, 141.1, 137.4, 135.4, 133.4, 132.6, 129.7 (2C), 128.5, 128.3 (2C), 126.0, 125.9, 124.0, 122.0, 121.6, 121.5, 112.8 (one carbon obscured by solvent).

HRMS (APCI) (m/z): found 317.0919 $[M+H]^+$ ($[C_{19}H_{12}N_2O_3 + H^+]^+$ requires 317.0921).

[NMR spectra] [table of contents]

***N*-(2,6-Dimethylbenzyl)-1-(1-nitro-2-phenyl-*9H*-carbazol-3-yl)methanamine (14a)**

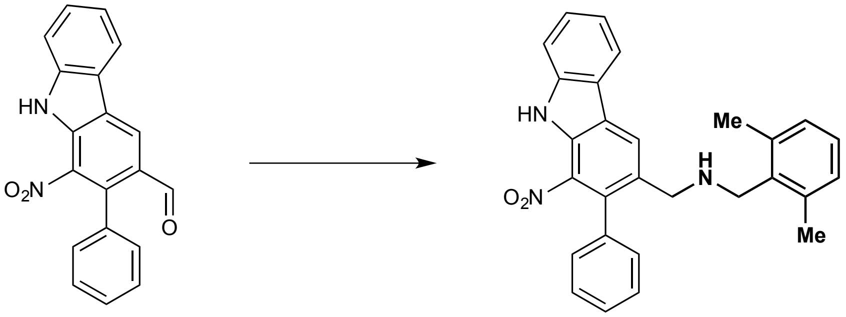

1-Nitro-2-phenyl-9*H*-carbazole-3-carbaldehyde (317 mg, 1.00 mmol, 1.0 equiv), sodium cyanoborohydride (75 mg, 1.20 mmol, 1.2 equiv), 3 Å molecular sieves (1.00 g, 1 g / mmol substrate), and 2,6-dimethylbenzylamine (270 mg, 2.00 mmol, 2.0 equiv) were suspended in THF (3.2 mL) and heated at 80 °C for 16 hours. The reaction mixture was cooled ,diluted with dichloromethane and washed with water and saturated aqueous NaCl solution before drying over magnesium sulphate, filtering, and concentrating *in vacuo* onto silica. Purification by column chromatography (10–30% ethyl acetate in 60–40 petroleum ether) yielded *N*-(2,6-dimethylbenzyl)-1-(1-nitro-2-phenyl-*9H*-carbazol-3-yl)methanamine (379 mg, 0.87 mmol, 87%) as a yellow solid.

m. p.: 104–106 °C.
R$_f$: 0.39 (30% ethyl acetate in 40–60 petroleum ether).
IR ν_{max}/cm^{-1} (film): 3055, 2922, 2868, 1608, 1528, 1496, 1467, 1335, 1322, 1289, 1205, 1180, 1086, 833, 770, 745, 698.
^{1}H NMR (500 MHz, acetonitrile-*d$_3$*) δ: 10.30 (br. s, 1H), 8.63 (s, 1H), 8.19 (d, *J* = 8.0 Hz, 1H), 7.68 (d, *J* = 8.0 Hz, 1H), 7.54 (t, *J* = 8.5 Hz, 1H), 7.49–7.43 (m, 3H), 7.36–7.30 (m, 3H), 7.00 (dd, *J* = 8.5, 6.0 Hz, 1H), 6.95 (d, *J* = 7.5 Hz, 2H), 3.65 (s, 2H), 3.59 (s, 2H), 2.25 (s, 6H), (N–H amine not observed).
^{13}C NMR (125 MHz, acetonitrile-*d$_3$*) δ: 141.7, 138.5, 138.2 (2C), 137.7, 135.7, 134.0, 133.6, 132.4, 129.7 (2C), 129.2 (2C), 128.9 (2C), 128.4 (2C), 127.9, 127.3, 126.7, 123.1, 121.8, 121.6, 112.9, 52.3, 48.1, 19.6 (2C).
HRMS (APCI) (m/z): found 436.2021 [M+H]$^+$ ([$C_{28}H_{25}N_3O_2$+H]$^+$ requires 436.2020).

[NMR spectra] [table of contents]

1-Mesityl-*N*-((1-nitro-2-phenyl-*9H*-carbazol-3-yl)methyl)methanamine (14b)

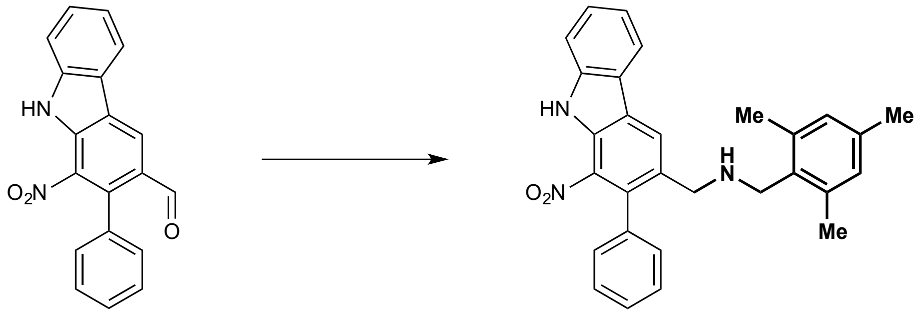

1-Nitro-2-phenyl-9*H*-carbazole-3-carbaldehyde (100 mg, 0.32 mmol, 1.0 equiv), sodium cyanoborohydride (24 mg, 0.38 mmol, 1.2 equiv), 3 Å molecular sieves (320 g, 1 g / mmol substrate), and mesitylmethanamine (95 mg, 0.64 mmol, 2.0 equiv) were suspended in THF (1.0 mL) and heated at 80 °C for 16 hours. The reaction mixture was cooled and diluted with dichloromethane and washed with water and saturated aqueous NaCl solution before drying over magnesium sulphate, filtering, and concentrating *in vacuo* onto silica. Purification by column chromatography (10–25% ethyl acetate in 60–40 petroleum ether) yielded 1-mesityl-*N*-((1-nitro-2-phenyl-9*H*-carbazol-3-yl)methyl)methanamine (110 mg, 0.25 mmol, 77%) as a yellow solid.

m.p.: 74–76 °C.
R$_f$: 0.42 (30% ethyl acetate in 40–60 petroleum ether).
IR ν_{max}/cm^{-1} (film): 3411, 2917, 2856, 1610, 1520, 1495, 1320, 1199, 1146, 850, 744, 700.
^{1}H NMR (500 MHz, acetonitrile-d_3) δ: 10.29 (s, 1H), 8.61 (app. d, J = 6.0 Hz, 1H), 8.17 (app. d, J = 8.0 Hz, 1H) 7.70–7.65 (m, 1H), 7.56–7.50 (m, 1H), 7.48–7.43 (m, 3H), 7.36–7.26 (m, 3H), 6.77 (s, 2H), 3.63 (s, 2H), 3.54 (s, 2H), 2.20 (s, 6H), 2.19 (s, 3H), (N–H amine not observed).
^{13}C NMR (125 MHz, acetonitrile-d_3) δ: 141.7, 138.5, 137.9, 137.1, 135.7, 134.6, 133.9, 132.4, 129.7 (2C), 129.5 (2C), 129.2, 129.1 (2C), 128.4 (2C), 127.1, 126.6, 126.0, 123.0, 121.7, 121.5, 112.8, 52.2, 47.8, 20.9, 19.5 (2C).
HRMS (APCI) (m/z): found 450.2179 [M+H]$^+$ ([$C_{29}H_{27}N_3O_2$+H]$^+$ requires 450.2176).

[NMR spectra] [table of contents]

N-(2,6-Dimethoxybenzyl)-1-(1-nitro-2-phenyl-$9H$-carbazol-3-yl)methanamine (14c)

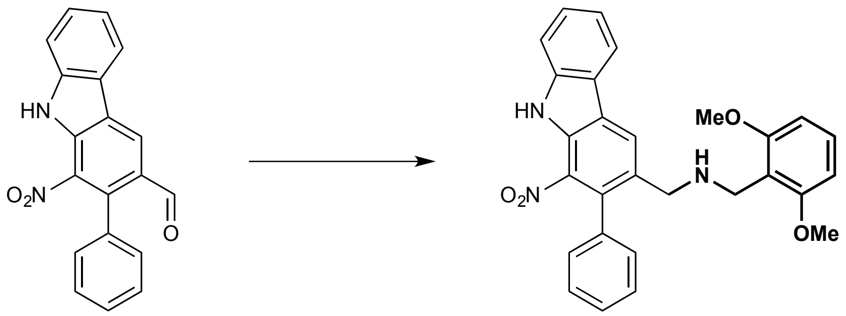

1-Nitro-2-phenyl-9*H*-carbazole-3-carbaldehyde (100 mg, 0.32 mmol, 1.0 equiv), sodium cyanoborohydride (24 mg, 0.38 mmol, 1.2 equiv), 3 Å molecular sieves (320 mg, 1 g / mmol substrate), and 2,6-dimethoxybenzylamine (107 mg, 0.64 mmol, 2.0 equiv) were suspended in THF (1.0 mL) and heated at 80 °C for 16 hours. The reaction mixture was cooled, diluted with dichloromethane and washed with water and saturated aqueous NaCl solution before drying over magnesium sulphate, filtering, and concentrating *in vacuo* onto silica. Purification by column chromatography (10–60% ethyl acetate in dichloromethane) yielded *N*-(2,6-dimethoxybenzyl)-1-(1-nitro-2-phenyl-*9H*-carbazol-3-yl)methanamine (87 mg, 0.19 mmol, 59%) as a yellow solid.

m.p.: 85–87 °C.

IR ν_{max}/cm^{-1} (film): 3395, 2939, 2836, 1595, 1520, 1474, 1321, 1247, 1199, 1118, 774, 744, 700.

^{1}H NMR (500 MHz, DMSO-d_6) δ: 10.32 (s, 1H), 8.58 (s, 1H), 8.16 (d, *J* = 7.5 Hz, 1H), 7.67 (dt, *J* = 8.0, 0.5 Hz, 1H), 7.53 (app. ddd, *J* = 8.5, 7.5, 1.0 Hz, 1H), 7.43–7.38 (m, 3H), 7.32 (app. ddd, *J* = 8.0, 7.0, 0.5 Hz, 1H), 7.24–7.20 (m, 2H), 7.17 (t, *J* = 8.0 Hz, 1H), 6.55 (d, *J* = 8.5 Hz, 2H), 3.71 (s, 6H), 3.69 (s, 2H), 3.51 (s, 2H), (N–H amine not observed).

^{13}C NMR (125 MHz, acetonitrile-d_3) δ: 159.5 (2C), 141.7, 138.3, 135.6, 133.8, 133.5, 131.9, 129.6, 129.4 (2C), 129.1 (2C), 128.3 (2C), 126.9, 126.6, 123.0, 121.7, 121.5, 116.3, 112.8, 104.7 (2C), 56.4 (2C), 51.1, 41.7.

HRMS (n-ESI) (m/z): found 468.1910 $[M+H]^+$ ($[C_{28}H_{25}N_3O_4+H]^+$ requires 468.1918).

[NMR spectra] [table of contents]

2-(2,6-Dimethylbenzyl)-5-nitro-4-phenyl-3,6-dihydropyrrolo[3,4-c]carbazol-1(*2H*)-one (15a)

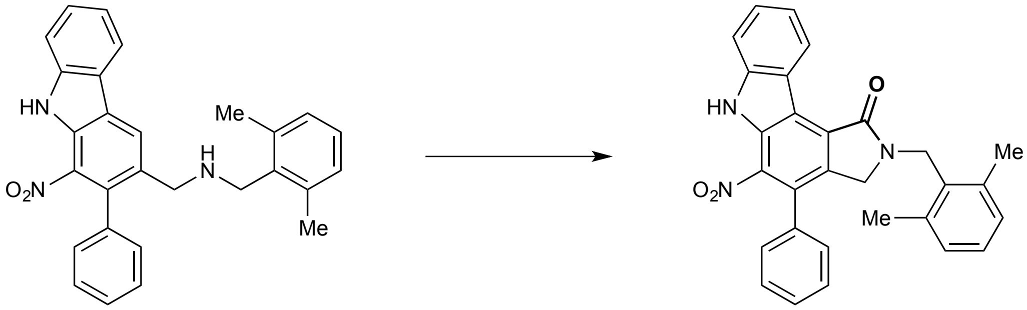

N-(2,6-Dimethylbenzyl)-1-(1-nitro-2-phenyl-*9H*-carbazol-3-yl)methanamine (110 mg, 0.253 mmol), copper acetate (23.0 mg, 0.127 mmol, 0.5 equiv) and palladium acetate (8.5 mg, 0.038 mmol, 0.15 equiv) was suspended in toluene (5.0 mL) under an atmosphere of CO (balloon). A balloon of air was added and the mixture was heated at 110 °C for 2 hours. The reaction mixture was cooled, filtered through Celite and concentrated onto silica. Column chromatography (10–60% ethyl acetate in 60–40 petroleum ether) afforded 2-(2,6-dimethylbenzyl)-5-nitro-4-phenyl-2,3-dihydropyrrolo[3,4-c]carbazol-1(*6H*)-one (106 mg, 0.23 mmol, 91%) as a yellow solid.
The product could be isolated in a yellow solid (21 mg, 0.048 mmol, 96%) if performed on a 0.050 mmol scale.

m.p.: 260 °C (decomposition).
$\mathbf{R_f}$: 0.36 (20% ethyl acetate/40–60 petroleum ether).
IR ν_{max}/cm^{-1} (film): 3423, 2960, 2923, 2853, 1691, 1519, 1496, 1455, 1345, 1303, 1201, 1148, 1084, 1026, 1011, 799, 761, 708,.
^{1}H NMR (500 MHz, acetonitrile-d_3) δ: 10.57 (br. s, 1H), 9.33 (d, *J* = 8.0 Hz, 1H), 7.74 (d, *J* = 8.4 Hz, 1H), 7.62 (app. td, *J* = 7.2, 1.2 Hz, 1H), 7.47–7.39 (m, 4 H), 7.30–7.28 (m, 2H), 7.09 (app. dd, *J* = 8.0, 6.7 Hz, 1H), 7.02 (app. d, *J* = 7.3 Hz, 2H), 4.91 (s, 2H), 3.88 (s, 2H), 2.30 (s, 6H).
^{13}C NMR (125 MHz, acetonitrile-d_3) δ: 168.0, 142.2, 138.9 (2C), 136.7, 135.5, 134.5, 134.4, 133.4, 131.5, 130.9, 129.7 (2C), 129.4 (2C), 129.3, 129.1, 128.9, 128.9 (2C), 127.2, 122.9, 122.1, 122.1, 112.7, 49.7, 41.4, 20.1 (2C).
HRMS (APCI) (m/z): found 462.1814 $[M+H]^+$ ($[C_{29}H_{23}N_3O_3+H]^+$ requires 462.1812).

[NMR spectra] [table of contents]

5-Nitro-4-phenyl-2-(2,4,6-trimethylbenzyl)-3,6-dihydropyrrolo[3,4-c]carbazol-1(*2H*)-one (15b)

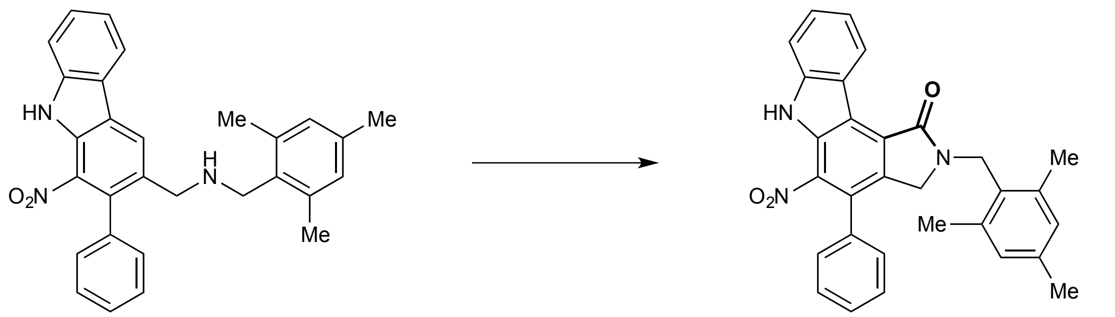

1-Mesityl-*N*-((1-nitro-2-phenyl-9*H*-carbazol-3-yl)methyl)methanamine (22.5 mg, 0.050 mmol), copper acetate (4.5 mg, 0.025 mmol, 0.5 equiv) and palladium acetate (1.8 mg, 0.008 mmol, 0.15 equiv) was suspended in toluene (2.0 mL) under an atmosphere of CO (balloon). A balloon of air was added and the mixture was heated at 110 °C for 2 hours. The reaction mixture was cooled, filtered through Celite and concentrated onto silica. Column chromatography (10–60% ethyl acetate in 60–40 petroleum ether) afforded 5-nitro-4-phenyl-2-(2,4,6-trimethylbenzyl)-3,6-dihydropyrrolo[3,4-c]carbazol-1(*2H*)-one (15 mg, 0.032 mmol, 63%) as a yellow solid.

m.p.: 240 °C (decomposition).

IR ν_{max}/cm^{-1} (film): 3421, 2922, 1686, 1516, 1327, 1301, 1197, 1150, 1012, 951, 902, 813, 754, 720, 699.

^{1}H NMR (500 MHz, acetonitrile-d_3) δ: 10.56 (s, 1H), 9.33 (d, *J* = 10.0 Hz, 1H), 7.74 (d, *J* = 10.0 Hz, 1H), 7.61 (app. ddd, *J* = 10.5, 9.0, 1.0 Hz, 1H), 7.48–7.38 (m, 4H), 7.31–7.26 (m, 2H), 6.85 (s, 2H), 4.86 (s, 2H), 3.87 (s, 2H), 2.25 (s, 6H), 2.21 (s, 3H).

^{13}C NMR (125 MHz, acetonitrile-d_3) δ: 167.9, 142.2, 138.7 (2C), 138.5, 136.7, 135.4, 134.4, 134.3, 131.5, 130.9, 130.3, 130.0 (2C), 129.7 (2C), 129.2, 129.0, 128.8 (2C), 127.2, 122.8, 122.0, 122.0, 112.6, 29.7, 41.1, 20.9, 20.0 (2C). CH_2Cl_2 impurity.

HRMS (ESI) (m/z): (APCI) (m/z): found 476.1970 $[M+H]^+$ ($[C_{30}H_{25}N_3O_3+H]^+$ requires 476.1969).

[NMR spectra] [table of contents]

2-(2,6-Dimethoxybenzyl)-5-nitro-4-phenyl-3,6-dihydropyrrolo[3,4-c]carbazol-1(*2H*)-one (15c)

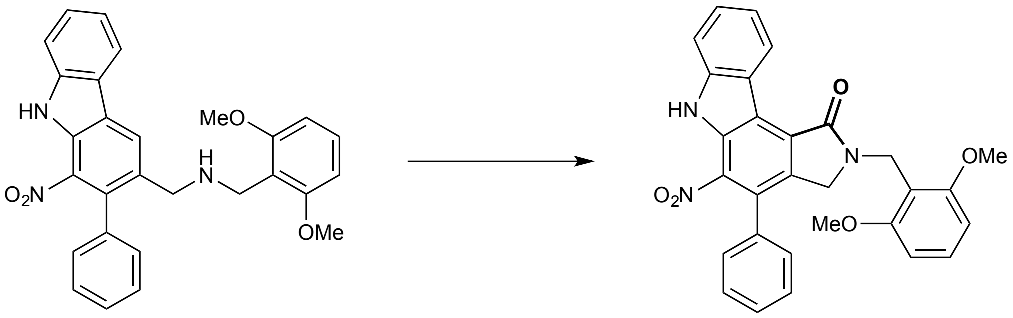

N-(2,6-Dimethoxybenzyl)-1-(1-nitro-2-phenyl-*9H*-carbazol-3-yl)methanamine (23.5 mg, 0.050 mmol), copper acetate (4.5 mg, 0.025 mmol, 0.5 equiv) and palladium acetate (1.8 mg, 0.008 mmol, 0.15 equiv) was suspended in toluene (2.0 mL) under an atmosphere of CO (balloon). A balloon of air was added and the mixture was heated at 110 °C for 2 hours. The reaction mixture was cooled, filtered through Celite and concentrated onto silica. Column chromatography (10–60% ethyl acetate in 60–40 petroleum ether) afforded 5-nitro-4-phenyl-2-(2,4,6-trimethylbenzyl)-3,6-dihydropyrrolo[3,4-c]carbazol-1(*2H*)-one (12 mg, 0.026 mmol, 51%) as a yellow solid.

m.p.: 246–248 °C.

IR ν_{max}/cm^{-1} (film): 3243, 2923, 2851, 1667, 1597, 1526, 1455, 1316, 1286, 1255, 1220, 1118, 817, 754, 706.

^{1}H NMR (500 MHz, acetonitrile-d_3) δ: 10.57 (s, 1H), 9.33 (d, *J* = 8.0 Hz, 1H), 7.74 (app. d, *J* = 8.0 Hz, 1H), 7.60 (app. ddd, *J* = 8.5, 7.0, 1.0 Hz 1H), 7.49–7.42 (m, 3H), 7.40 (app. ddd, *J* = 8.0, 7.0, 1.0 Hz, 1H), 7.33–7.29 (m, 2H), 7.26 (t, *J* = 8.5 Hz, 1H), 6.61 (d, *J* = 8.5 Hz, 2H), 4.86 (s, 2H), 3.98 (s, 2H), 3.72 (s, 6H).

^{13}C NMR (125 MHz, acetonitrile-d_3) δ: 167.7, 160.1 (2C), 142.2, 137.0, 135.6, 134.6, 132.1, 131.0 130.8, 129.7 (2C), 129.2, 129.0, 128.9 (2C), 127.3, 122.8, 122.1, 122.0, 112.9 (2C) 112.6, 105.1 (2C), 56.5 (2C), 50.5, 35.7.

HRMS (ESI) (m/z): (n-ESI) (m/z): found 494.1701 $[M+H]^+$ ($[C_{29}H_{23}N_3O_5+H]^+$ requires 494.1710).

[NMR spectra] [table of contents]

6-(2,6-Dimethylbenzyl)-6,7,12,13-tetrahydro-5*H*-indolo[2,3-a]pyrrolo[3,4-c]carbazol-5-one (16)

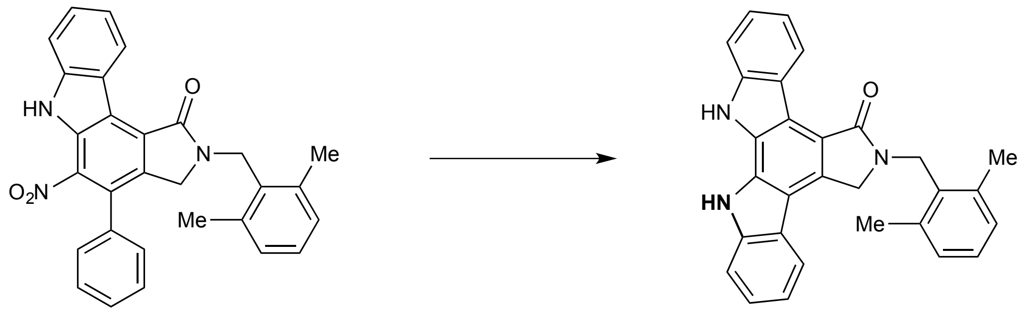

A suspension of 2-(2,6-dimethylbenzyl)-5-nitro-4-phenyl-2,3-dihydropyrrolo[3,4-c]carbazol-1(*6H*)-one (50 mg, 0.11 mmol) in freshly distilled triethyl phosphite (1.0 mL) was subjected to microwave irradiation with a ceiling temperature of 210 °C for 20 minutes. The crude mixture was allowed to cool and precipitate and hexane was added before filtration. The resulting solid was then washed three times with hexane and filtered through silica (10% methanol in dichloromethane) to afford 6-(2,6-Dimethylbenzyl)-6,7,12,13-tetrahydro-5*H*-indolo[2,3-a]pyrrolo[3,4-c]carbazol-5-one (40 mg, 0.09 mmol, 86%) as an off-white solid.

m.p.: 280 °C (decomposition).
R_f: 0.32 (40% ethyl acetate/40–60 petroleum ether).
IR ν_{max}/cm^{-1} (film): 3340, 2921, 2852, 1647, 1585, 1457, 1413, 1398, 1329, 1223, 1116, 1012, 965, 774, 739, 670.
^{1}H NMR (500 MHz, DMSO-d_6) δ: 11.47 (s, 1H), 11.32 (s, 1H), 9.30 (d, J = 8.0 Hz, 1H), 7.83 (d, J = 8.0 Hz, 1H), 7.75 (t, J = 9.2 Hz, 2H), 7.45 (t, J = 7.6 Hz, 2H), 7.26 (t, J = 7.6 Hz, 2H), 7.19–7.06 (m, 3 H), 4.97 (s, 2H), 4.70 (s, 2H), 2.46 (s, 6H).
^{13}C NMR (125 MHz, acetonitrile-d_3) δ: 168.9, 139.2, 139.2, 137.6 (2C), 133.5, 130.0, 128.4 (2C), 127.8, 127.5, 125.5, 125.2 (2C), 125.1, 122.7, 122.3, 120.9, 120.0, 119.1, 118.2, 115.5, 113.8, 112.0, 111.4, 48.4, 39.7, 19.8 (2C).
HRMS (APCI) (m/z): found 430.1908 $[M+H]^+$ ($[C_{29}H_{23}N_3O+H]^+$ requires 430.1914).

[NMR spectra] [table of contents]

***Staurosporinone* (3)**

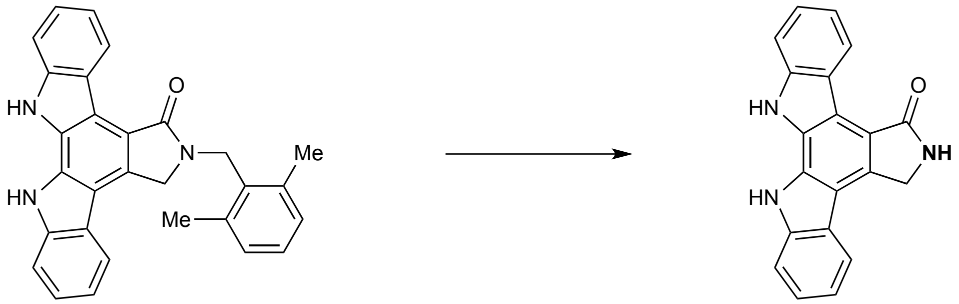

6-(2,6-Dimethylbenzyl)-6,7,12,13-tetrahydro-5H-indolo[2,3-a]pyrrolo[3,4-c]carbazol-5-one (5.0 mg, 11.6 μmol) and tetrabutylammonium iodide (21.5 mg, 58 μmol) were suspended in *ortho*-dichlorobenzene (1 mL) and boron trichloride (1 M in dichloromethane, 58 μL, 58 μmol) before subjection to microwave irradiation with a ceiling temperature of 210 °C for 30 minutes. The resulting solution was loaded directly on to silica and purification by column chromatography (25% acetone in dichloromethane) afforded *staurosporinone* (2.8 mg, 9.0 μmol, 78%) as an off-white solid.[4]

R_f: 0.59 (25% acetone/dichloromethane).

IR ν_{max}/cm^{-1} (film): 3315, 1647, 1579, 1456, 1393, 1331, 1259.

^{1}H NMR (400 MHz, 95:5 DMSO-d_6 / acetone-d_6) δ: 11.47 (br. s, 1H), 11.30 (br. s, 1H), 9.22 (d, J = 8.0 Hz, 1H), 8.27 (br. s, 1H), 8.04 (d, J = 8.0 Hz, 1H), 7.79 (d, J = 8.0 Hz, 1H), 7.72 (dd, J = 8.0, Hz, 1H), 7.48 (ddd, J = 8.0, 7.2, 0.8 Hz, 1H), 7.42 (ddd, J = 8.4, 7.2, 1.2 Hz, 1H), 7.31 (t, J = 7.6 Hz, 1H), 7.23 (t, J = 7.6 Hz, 1H), 4.96 (br. s, 2H).

^{13}C NMR (125 MHz, DMSO-d_6) δ: 172.4, 139.2, 139.1, 132.9, 127.9, 125.4, 125.2, 125.0 (2C), 122.8, 122.6, 121.1, 119.9, 119.0, 118.9, 115.6, 114.1, 111.9, 111.4, 45.3.

HRMS (APCI) (m/z): found 312.1132 $[M+H]^+$ ($[C_{20}H_{13}N_3O+H]^+$ requires 312.1131).

[NMR spectra] [table of contents]

4. Rajeshwaran, G. G.; Mohanakrishnan, A. K., *Org. Lett.* 2011, **13**, 1418

Table S1. Comparison of herein obtained and previously reported NMR shifts for staurosporinone.

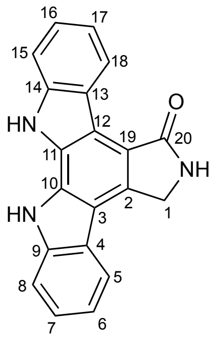

H atom	This work δ (p.p.m.)	Mohanakrishnan[a] δ (multiplicity) (p.p.m.)	Δδ (p.p.m.)
NH (carbazole)	11.47 (br. s, 1H)	11.52 (br. s, 1H)	0.05
NH (carbazole)	11.30 (br. s, 1H)	11.35 (br. s, 1H)	0.05
H18	9.22 (d, 1H)	9.24 (d, 1H)	0.02
NH (lactam)	8.27 (s, 1H)	8.51(s, 1H)	0.24
H5	8.04 (d, 1H)	8.06 (d, 1H)	0.02
H8	7.79 (d, 1H)	7.80 (d, 1H)	0.01
H15	7.72 (d 1H)	7.74 (d, 1H)	0.02
H7	7.31 (ddd, 1H)	7.52 - 7.41 (m, 2H)	
H16	7.23 (ddd, 1H)		
H6	7.31 (t, 1H)	7.32 (t, 1H)	0.01
H17	7.23 (t, 1H)	7.24 (t, 1H)	0.01
H1	4.96 (s, 2H)	4.96 (s, 2H)	0.00

[a]*Org. Lett.*, **2011**, *13*, 1418

C atom	This work δ (p.p.m.)	Mohanakrishnan[a] δ (p.p.m.)	Δδ (p.p.m.)
C20	172.4	172.4	0.0
C9	139.2	139.2	0.0
C14	139.1	139.1	0.0
C2	132.9	132.9	0.0
C10	127.9	127.8	0.1
C18	125.4	125.3	0.1
C16	125.2	125.2	0.0
C7	125.0	125.0	0.0
C11	125.0	125.0	0.0
C13	122.8	122.8	0.0
C4	122.6	122.5	0.1
C5	121.1	121.1	0.0
C6	119.9	119.9	0.0
C17	119.0	118.9	0.1
C19	118.9	118.8	0.1
C12	115.6	115.6	0.0
C3	114.1	114.1	0.0
C8	111.9	111.9	0.0
C15	111.4	111.3	0.1
C1	45.3	45.3	0.0

[a]*Org. Lett.*, **2011**, *13*, 1418

NMR SPECTRA

***N*,*N*-Dibenzyl-4-methylaniline (7)**

[experimental procedure] [table of contents]

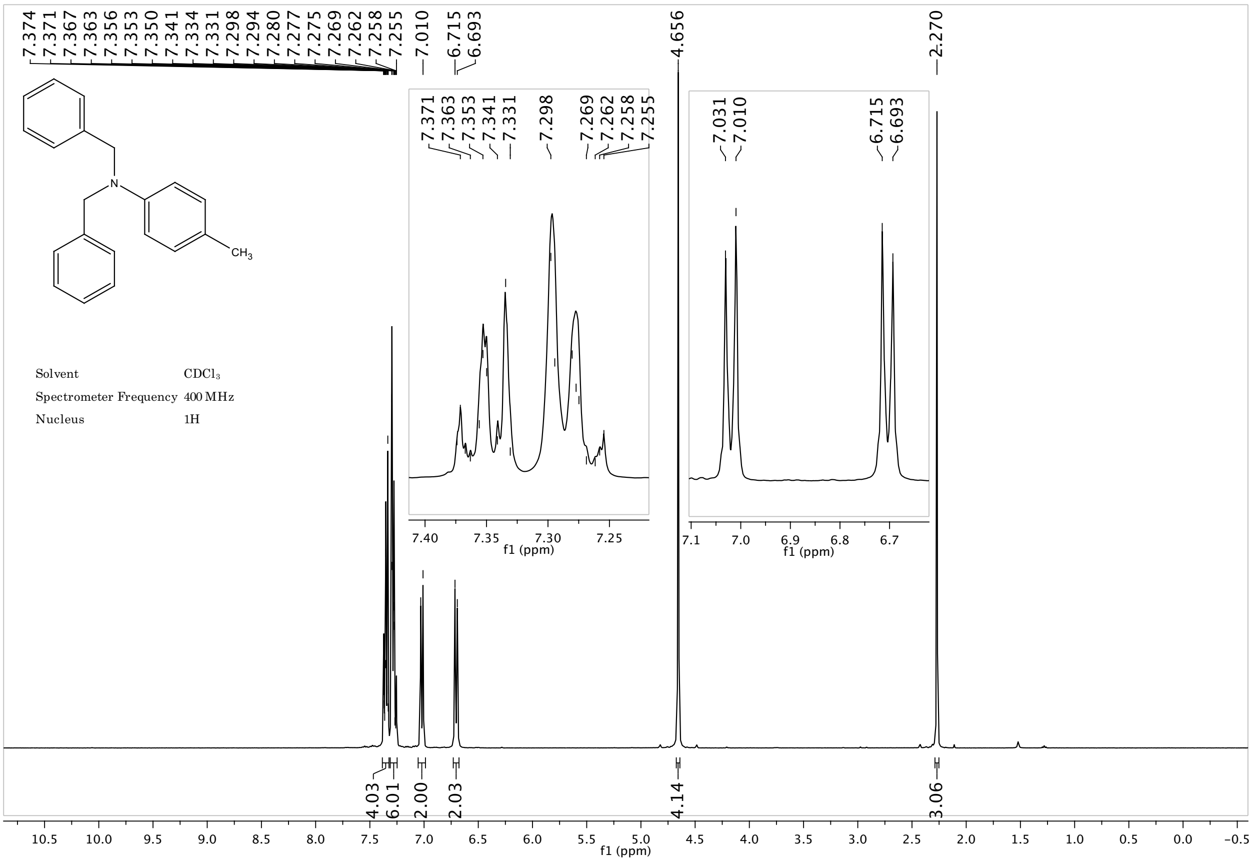

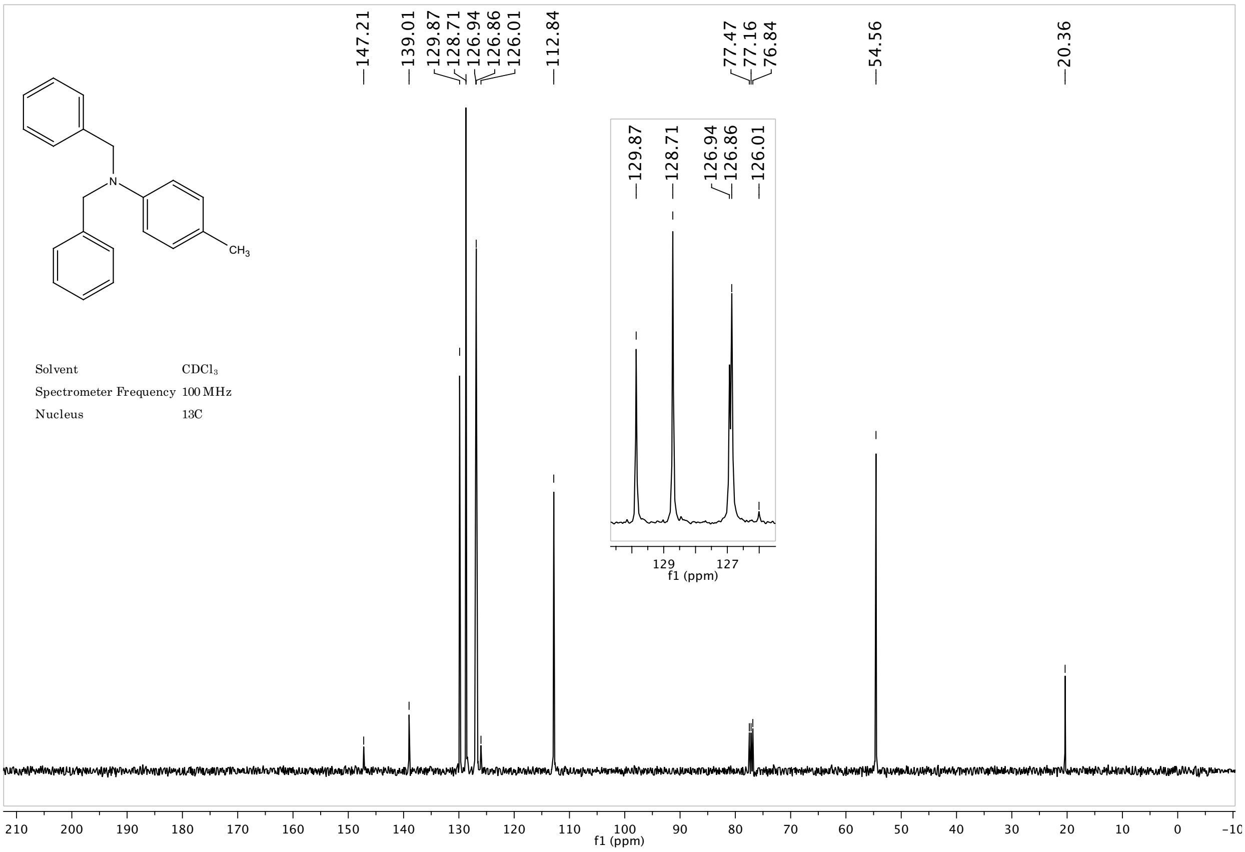

N,*N*-Dibenzyl-5-methyl-[1,1'-biphenyl]-2-amine (8)

[experimental procedure] [table of contents]

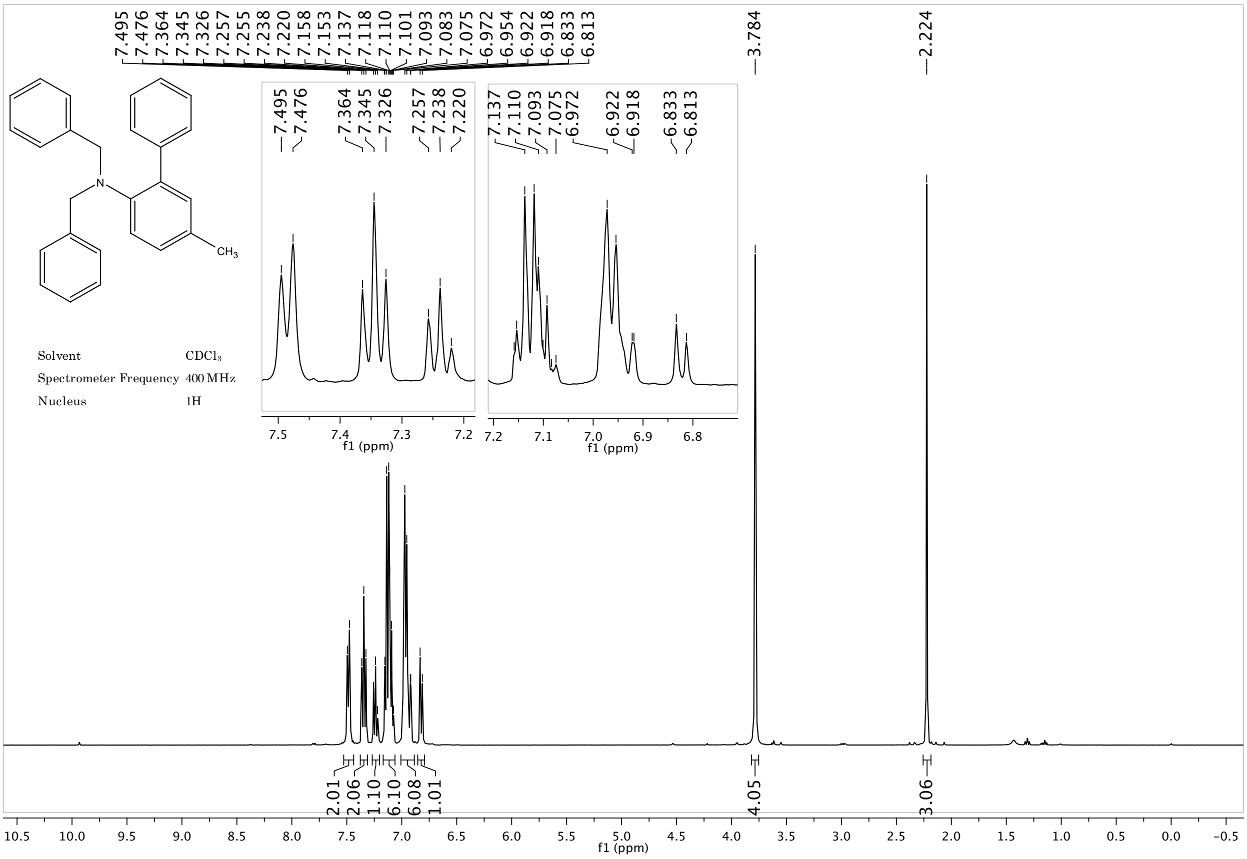

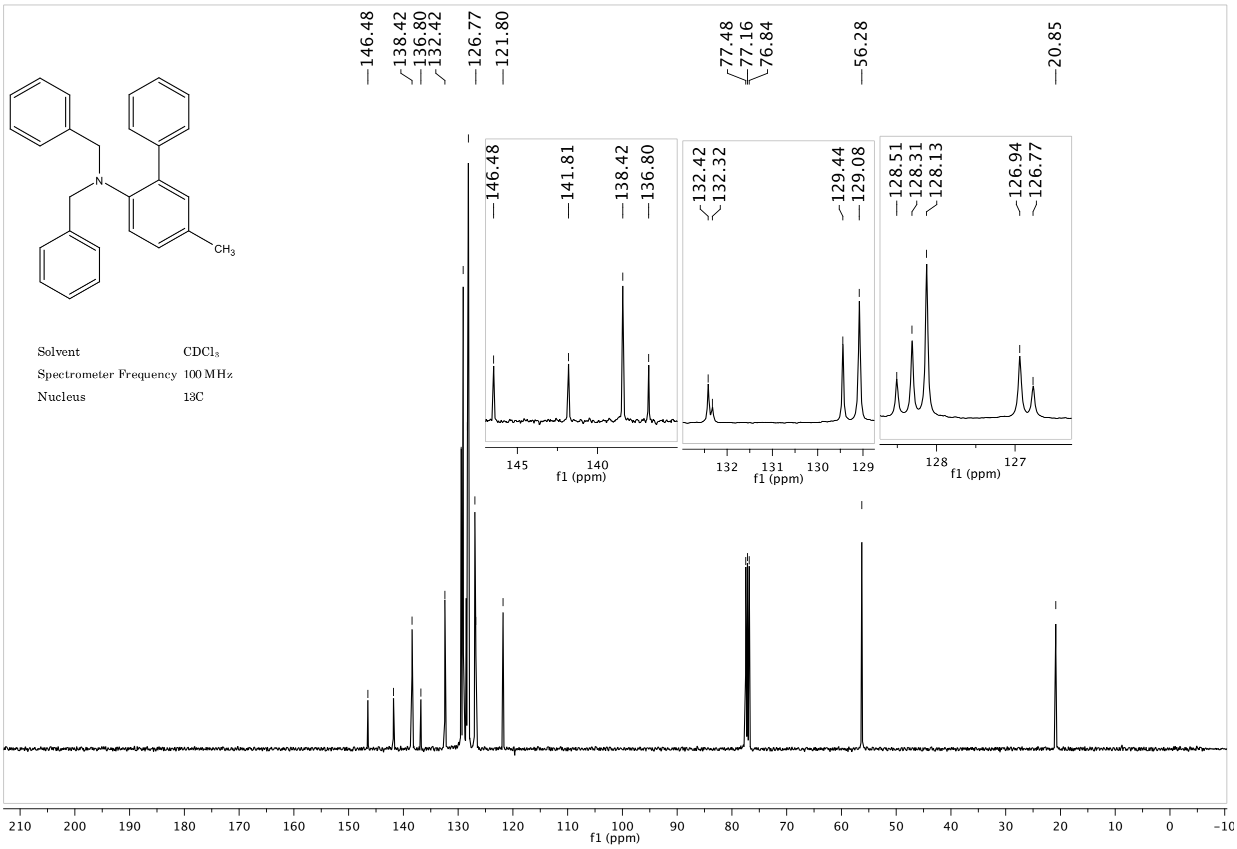

N-(5-Methyl-[1,1'-biphenyl]-2-yl)acetamide N-(5-Methyl-[1,1'-biphenyl]-2-yl)propionamide (9)

[experimental procedure] [table of contents]

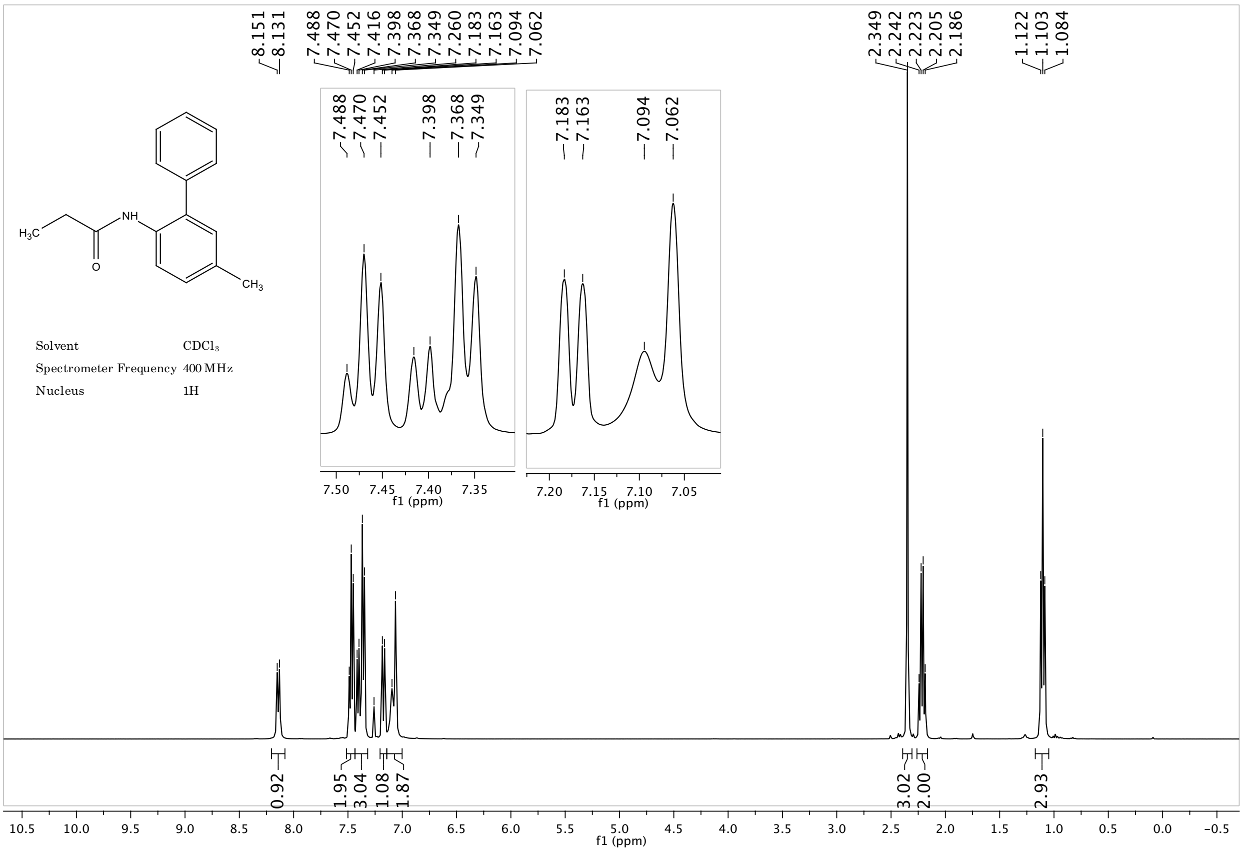

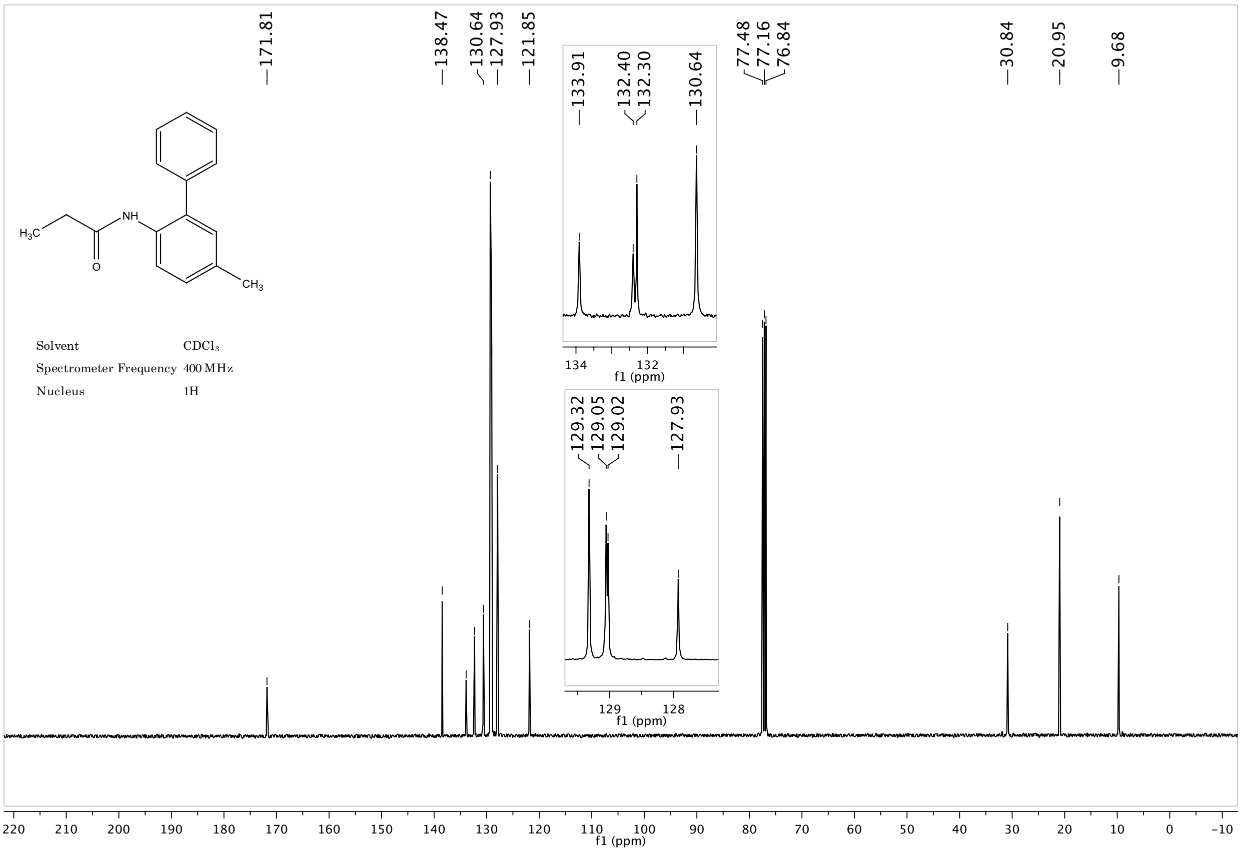

***N*-(5'-Methyl-[1,1':4',1''-terphenyl]-2'-yl)propionamide (10)**

[experimental procedure] [table of contents]

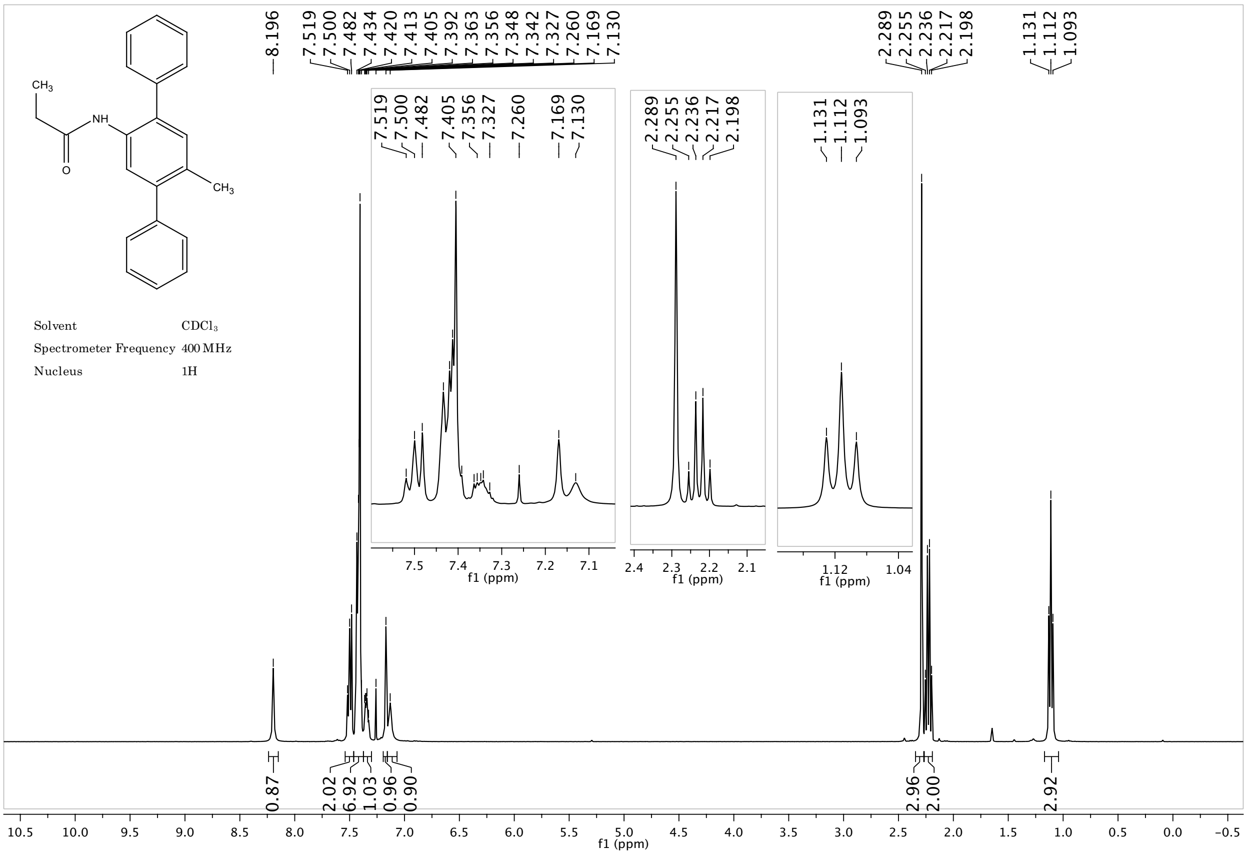

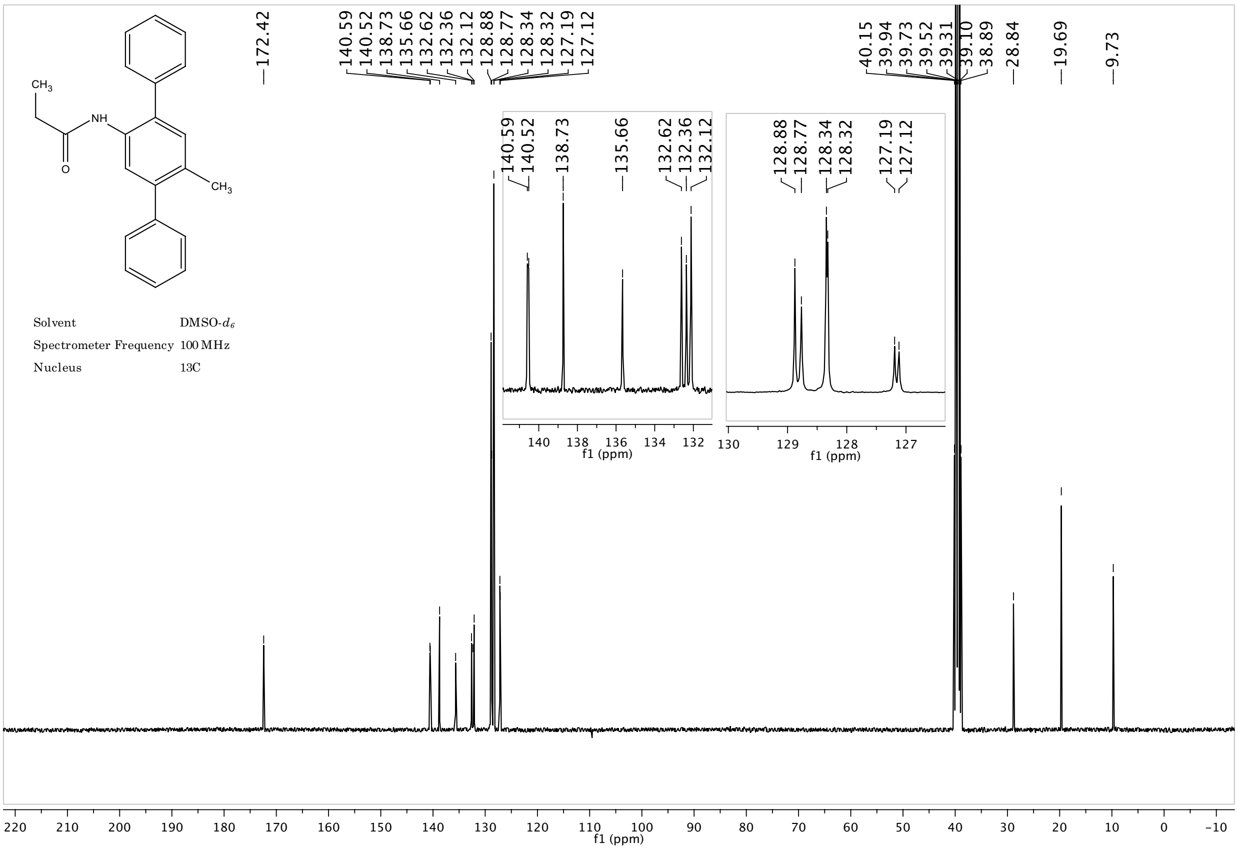

N-(5'-Methyl-3'-nitro-[1,1':4',1''-terphenyl]-2'-yl)propionamide (11)

[experimental procedure] [table of contents]

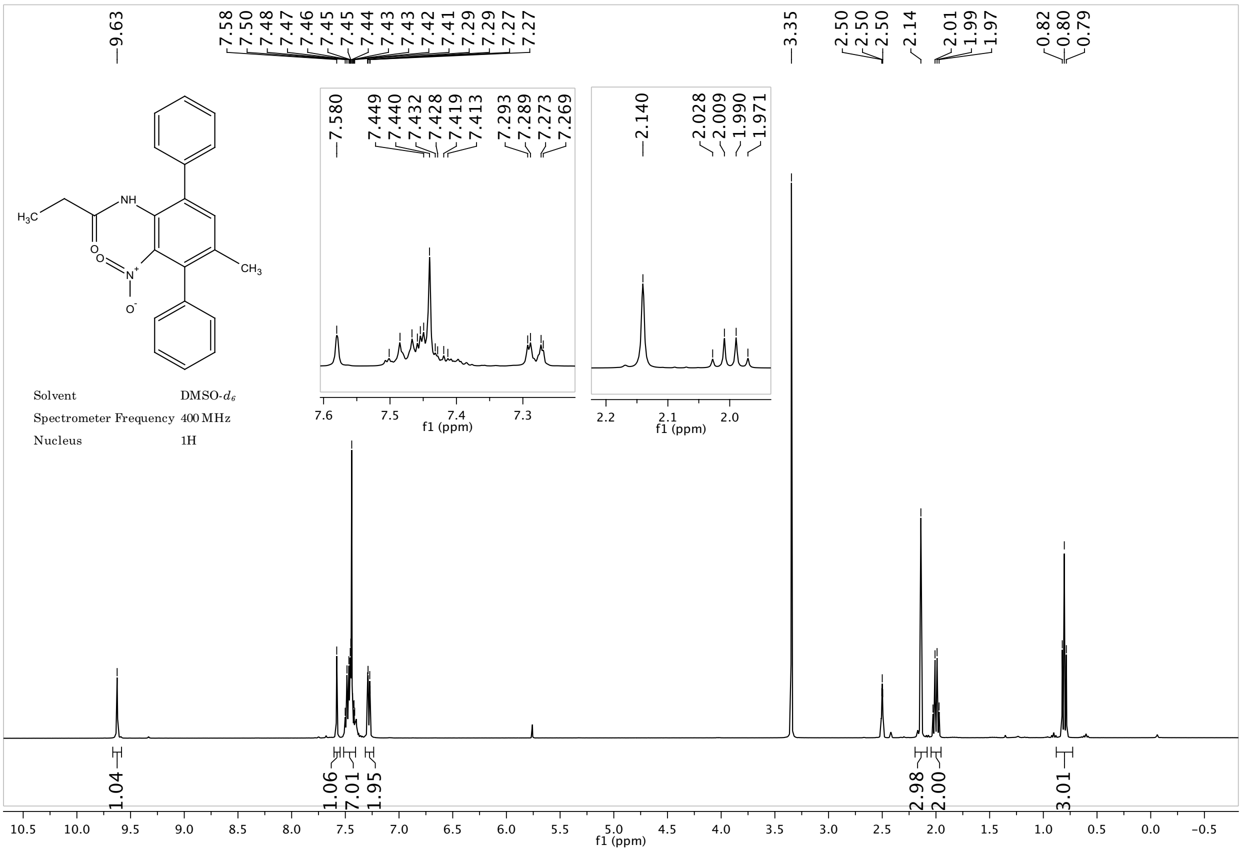

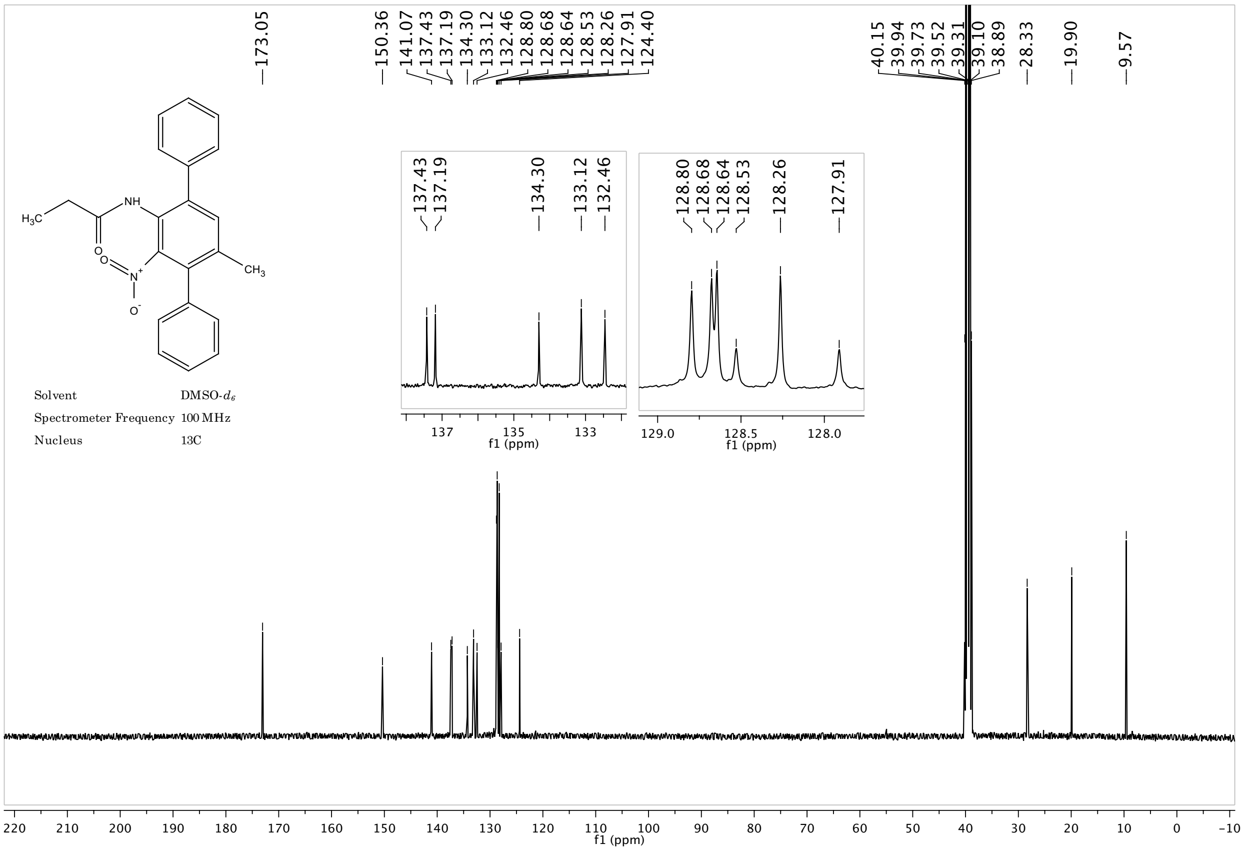

1-(3-Methyl-1-nitro-2-phenyl-9H-carbazol-9-yl)propan-1-one (12)

[experimental procedure] [table of contents]

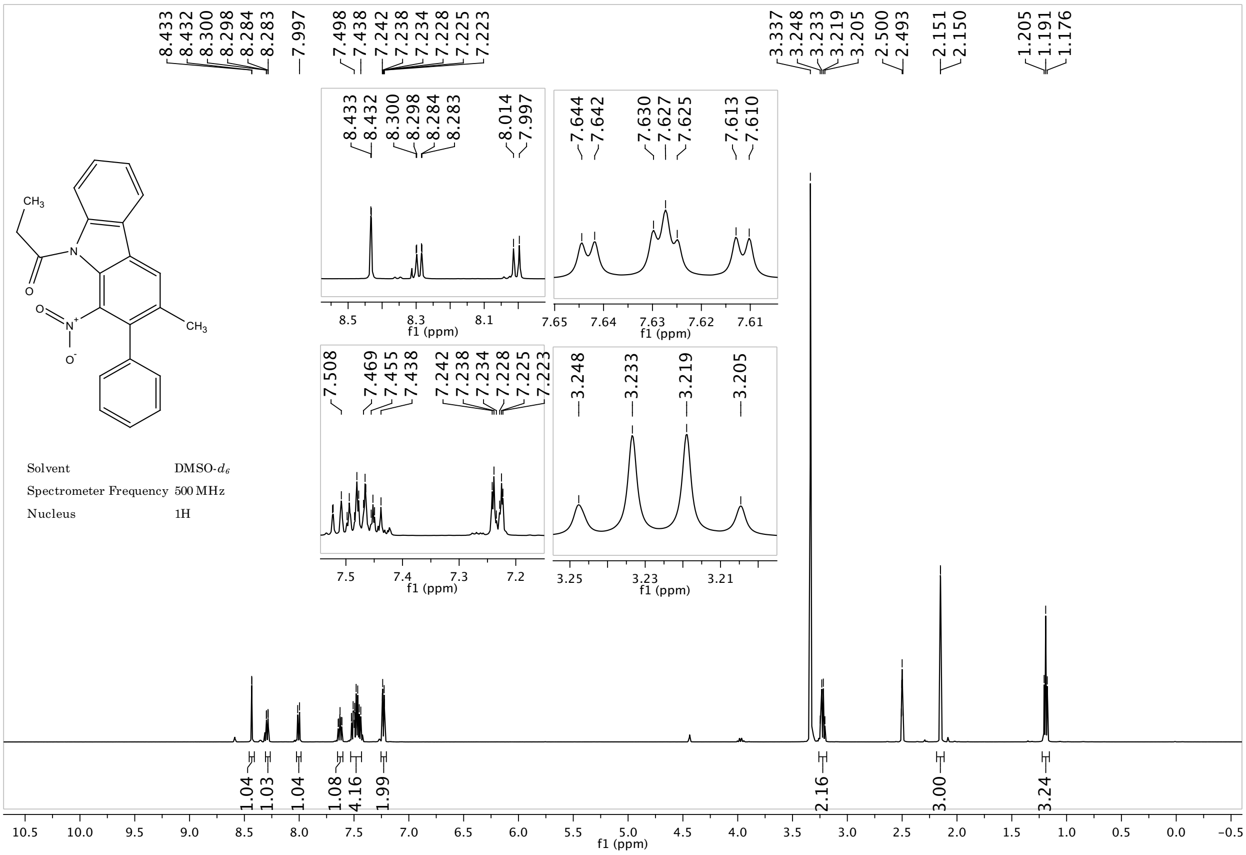

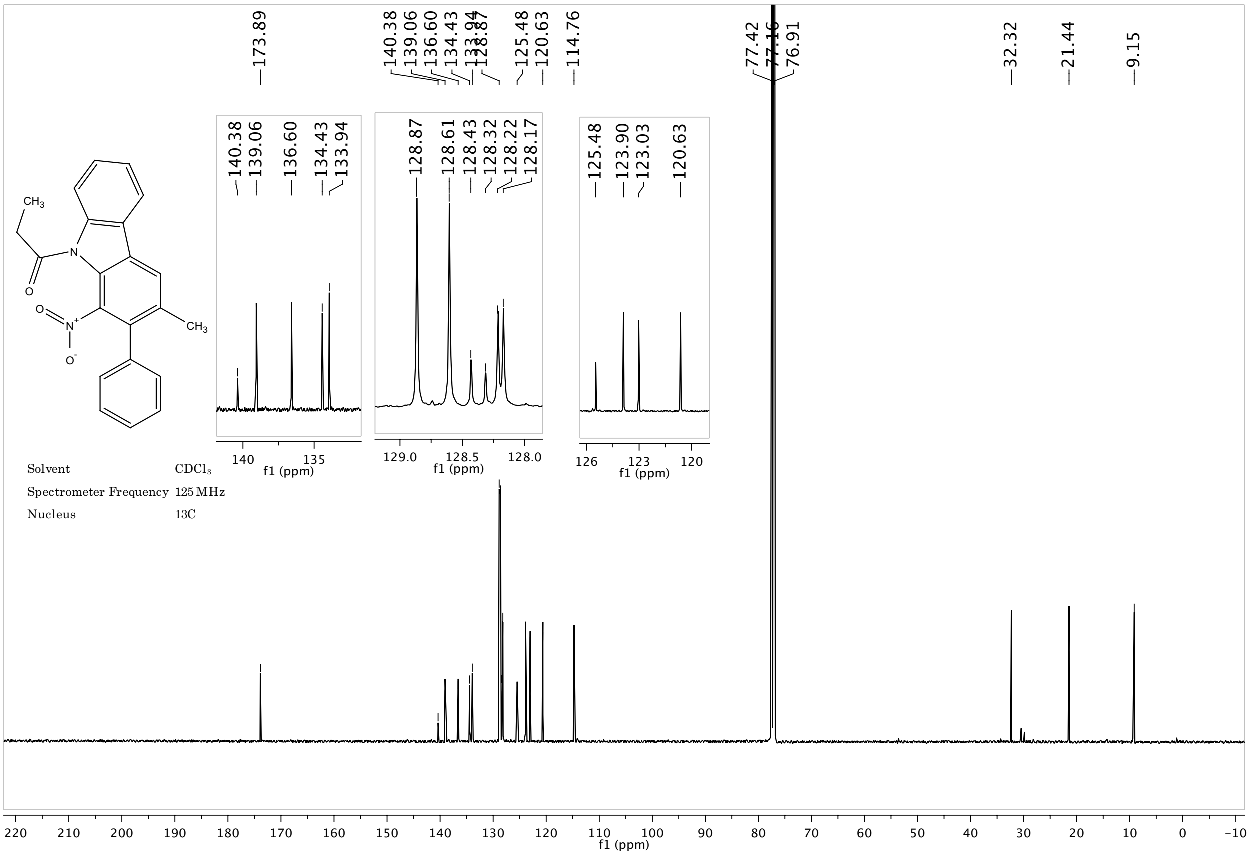

1-(3-(Dibromomethyl)-1-nitro-2-phenyl-9*H*-carbazol-9-yl)propan-1-one (12a)

[experimental procedure] [table of contents]

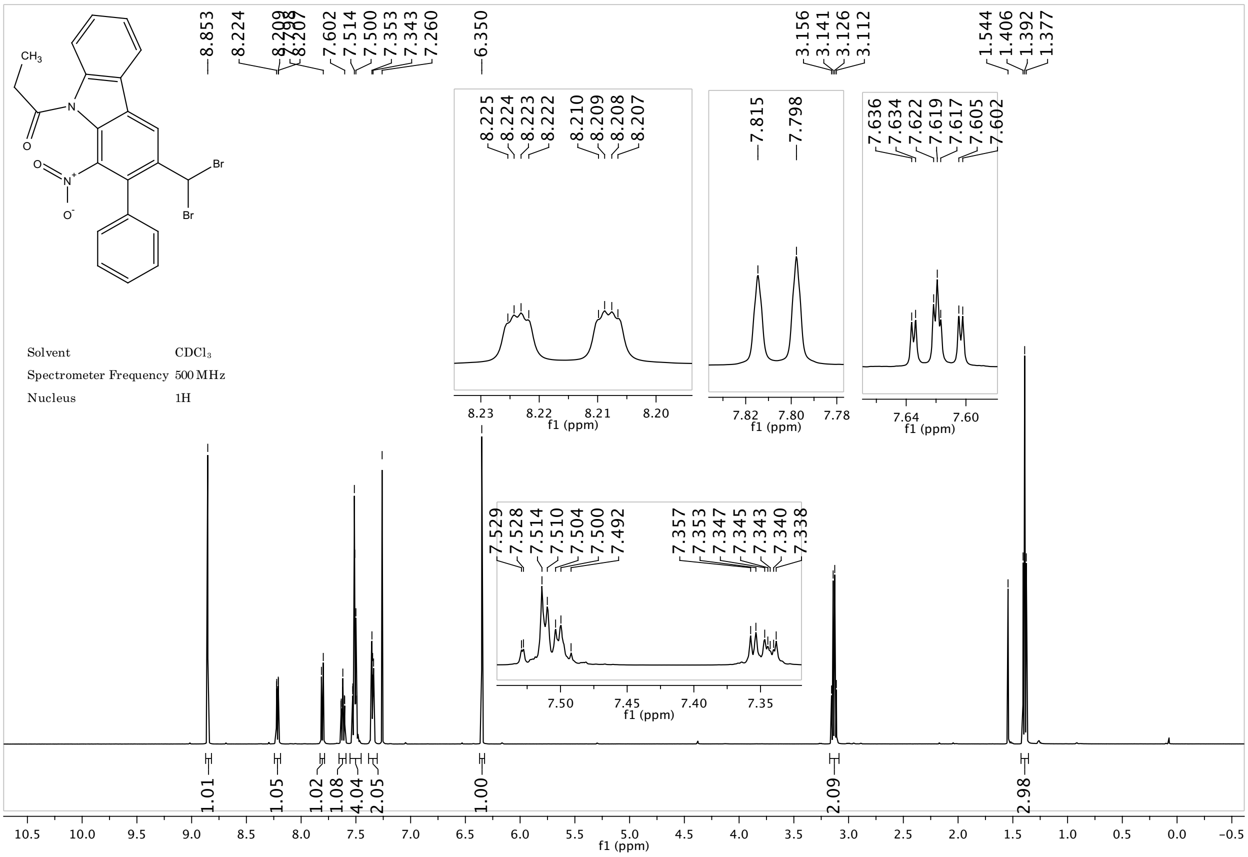

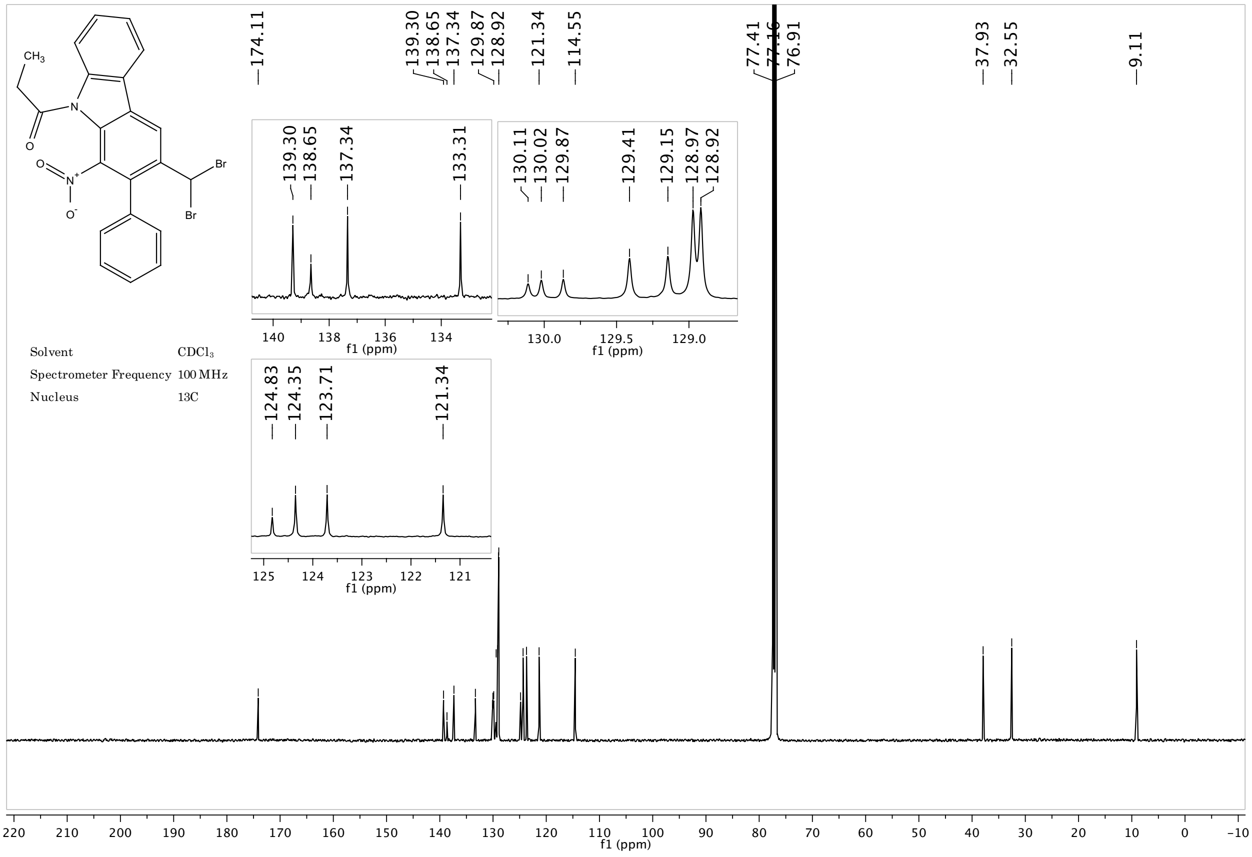

1-Nitro-2-phenyl-9*H*-carbazole-3-carbaldehyde (13)

[experimental procedure] [table of contents]

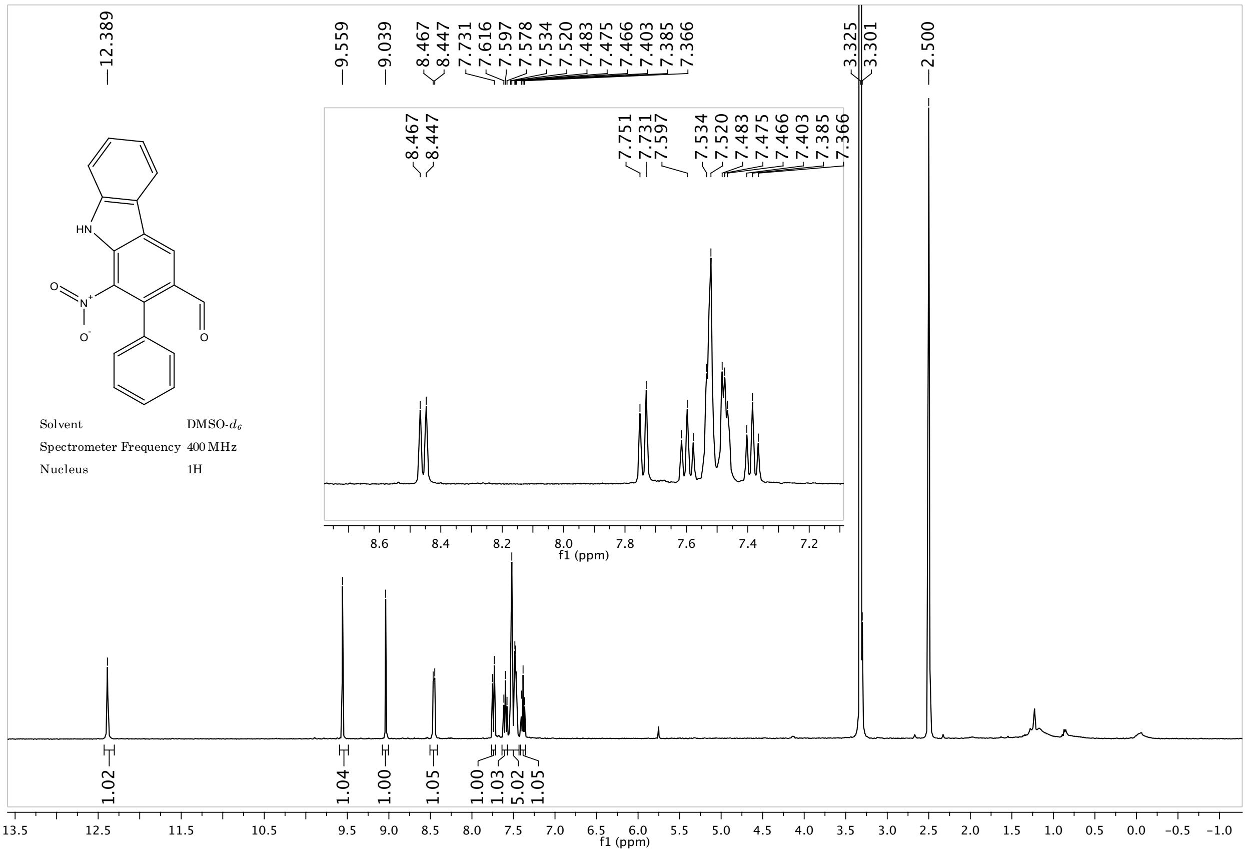

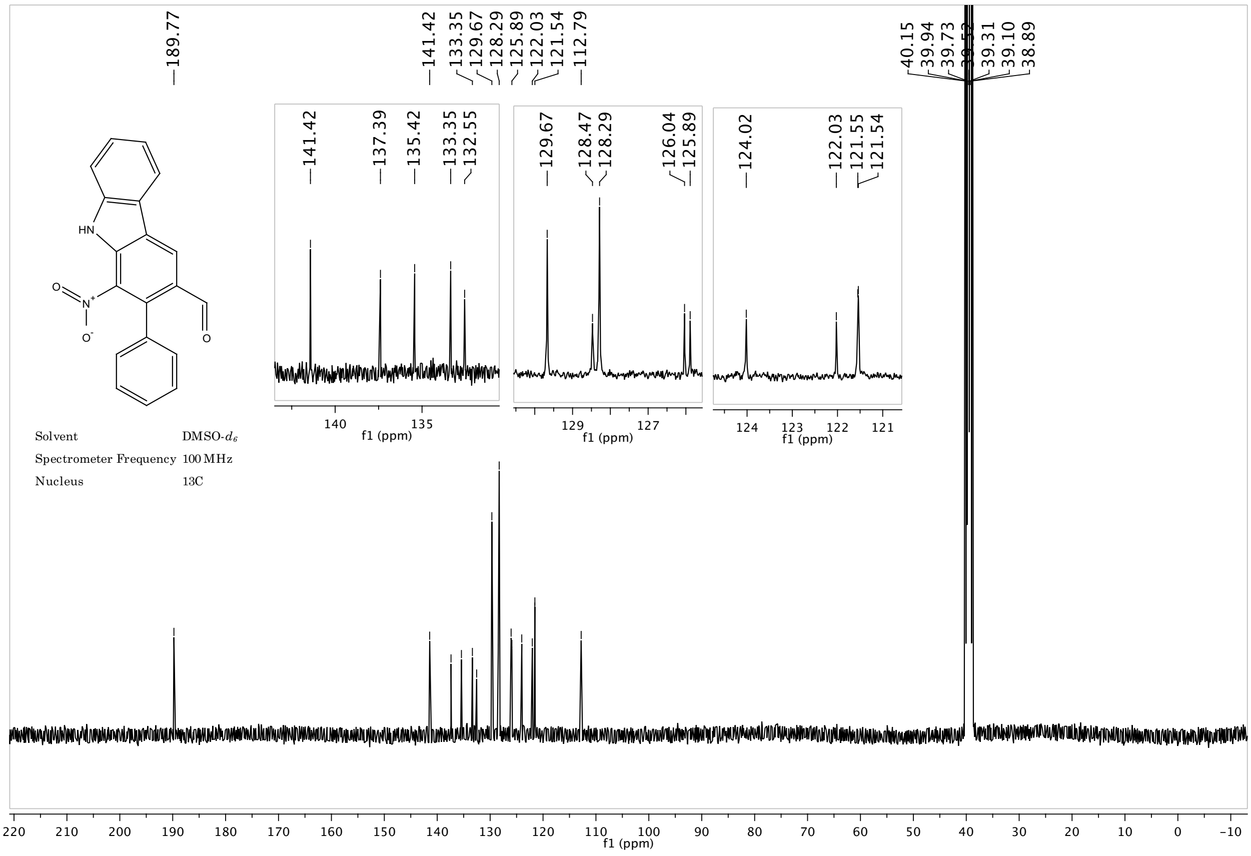

N-(2,6-Dimethylbenzyl)-1-(1-nitro-2-phenyl-9*H*-carbazol-3-yl)methanamine (14a)

[experimental procedure] [table of contents]

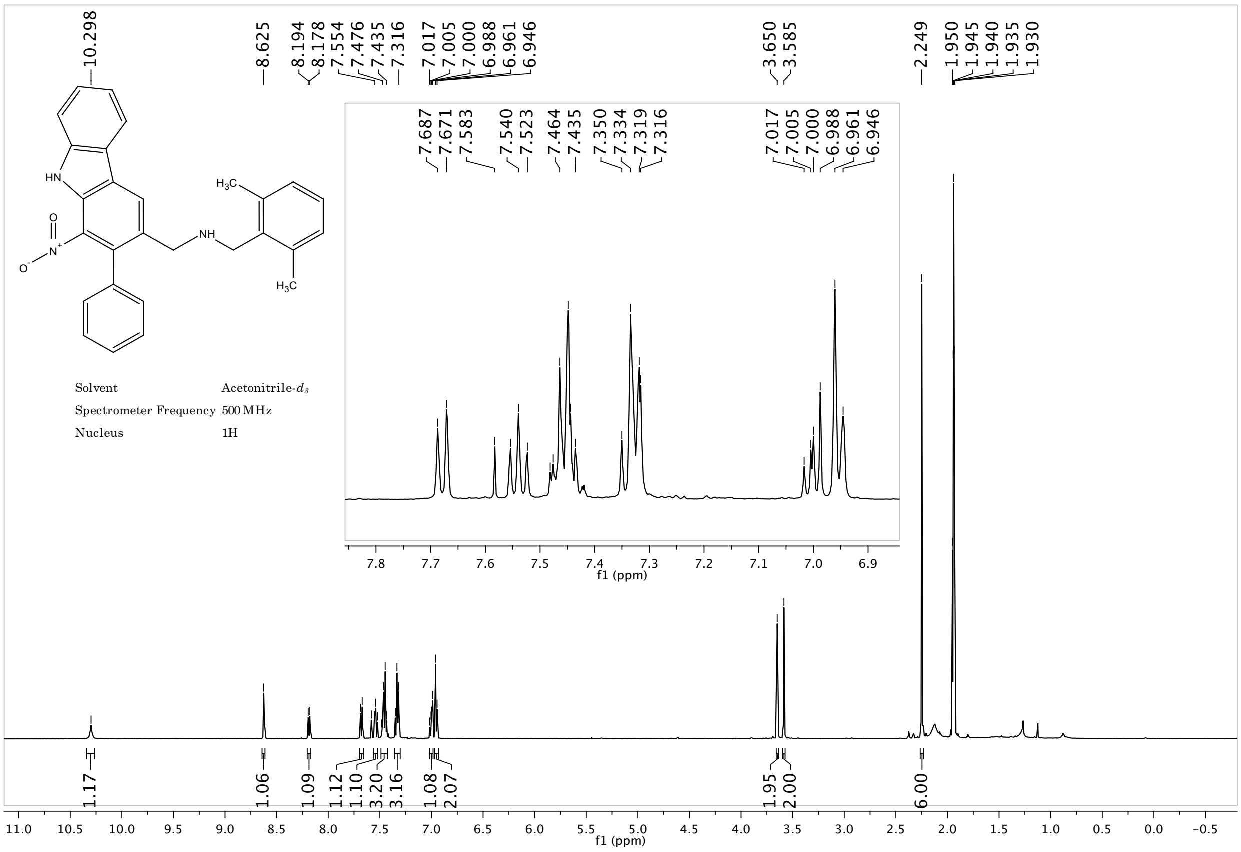

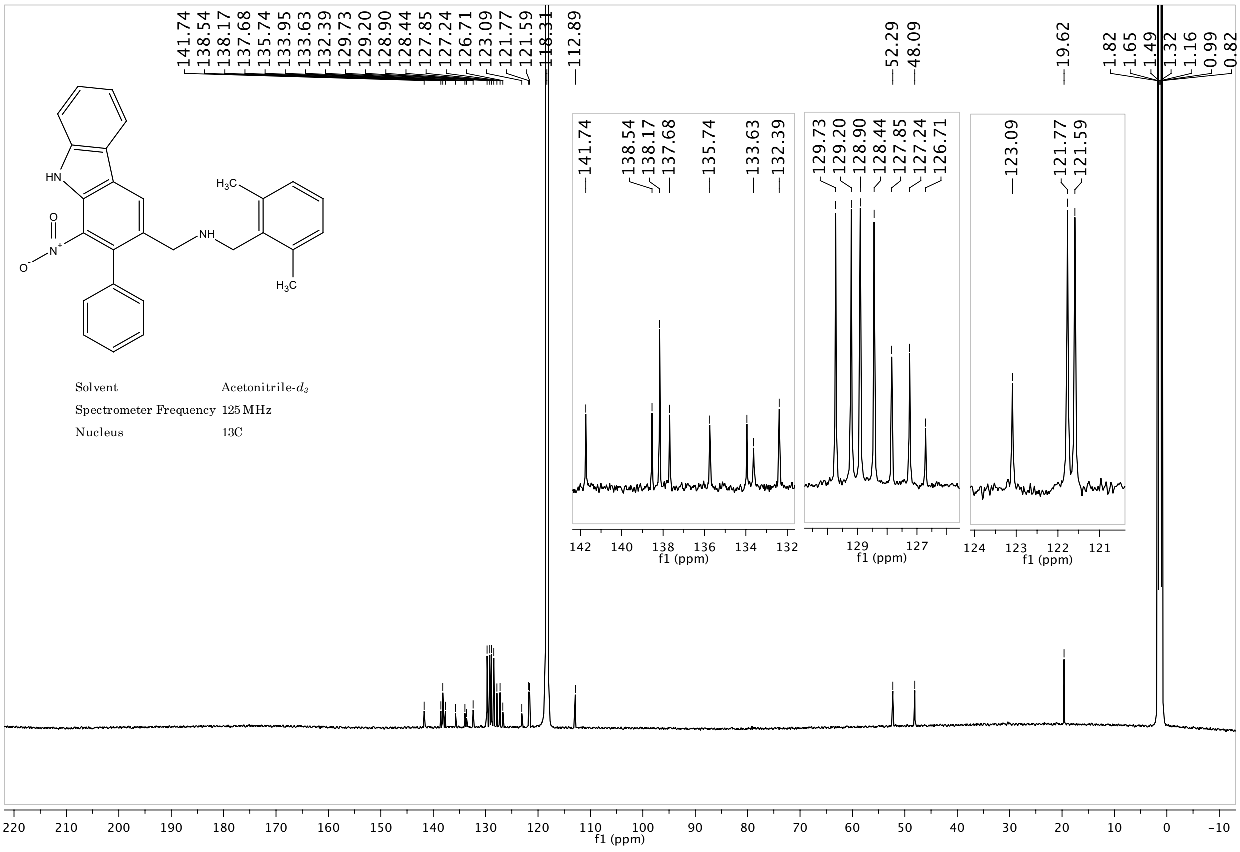

1-Mesityl-*N*-((1-nitro-2-phenyl-*9H*-carbazol-3-yl)methyl)methanamine (14b)

[experimental procedure] [table of contents]

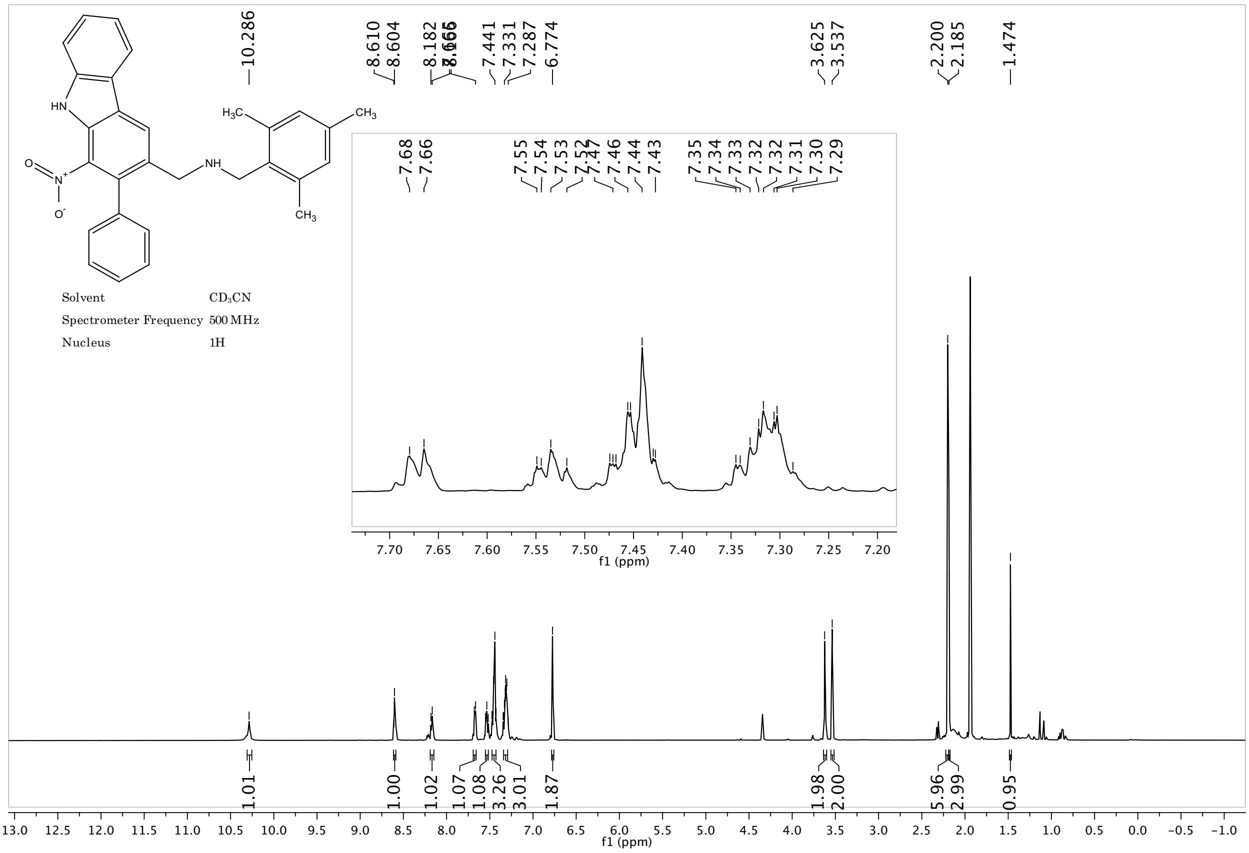

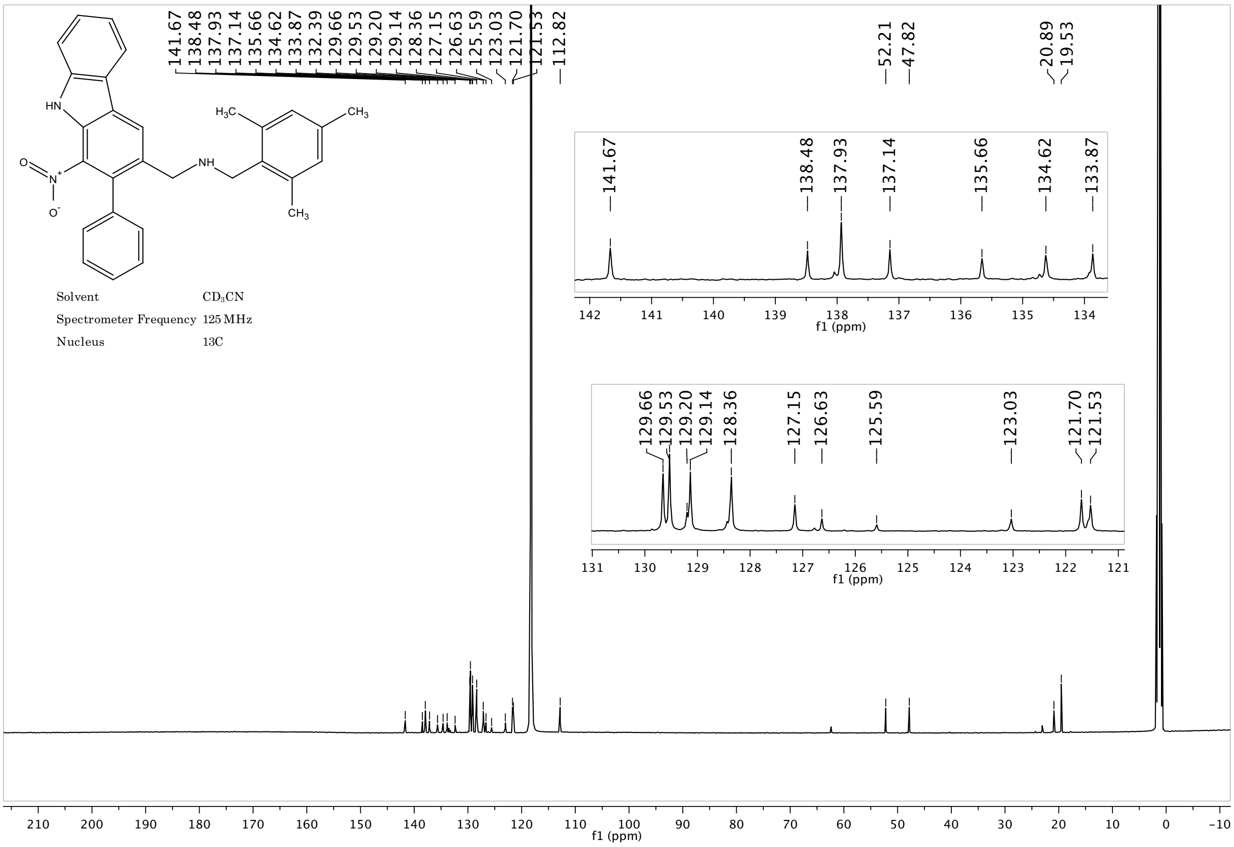

N-(2,6-Dimethoxybenzyl)-1-(1-nitro-2-phenyl-*9H*-carbazol-3-yl)methanamine (14c)

[experimental procedure] [table of contents]

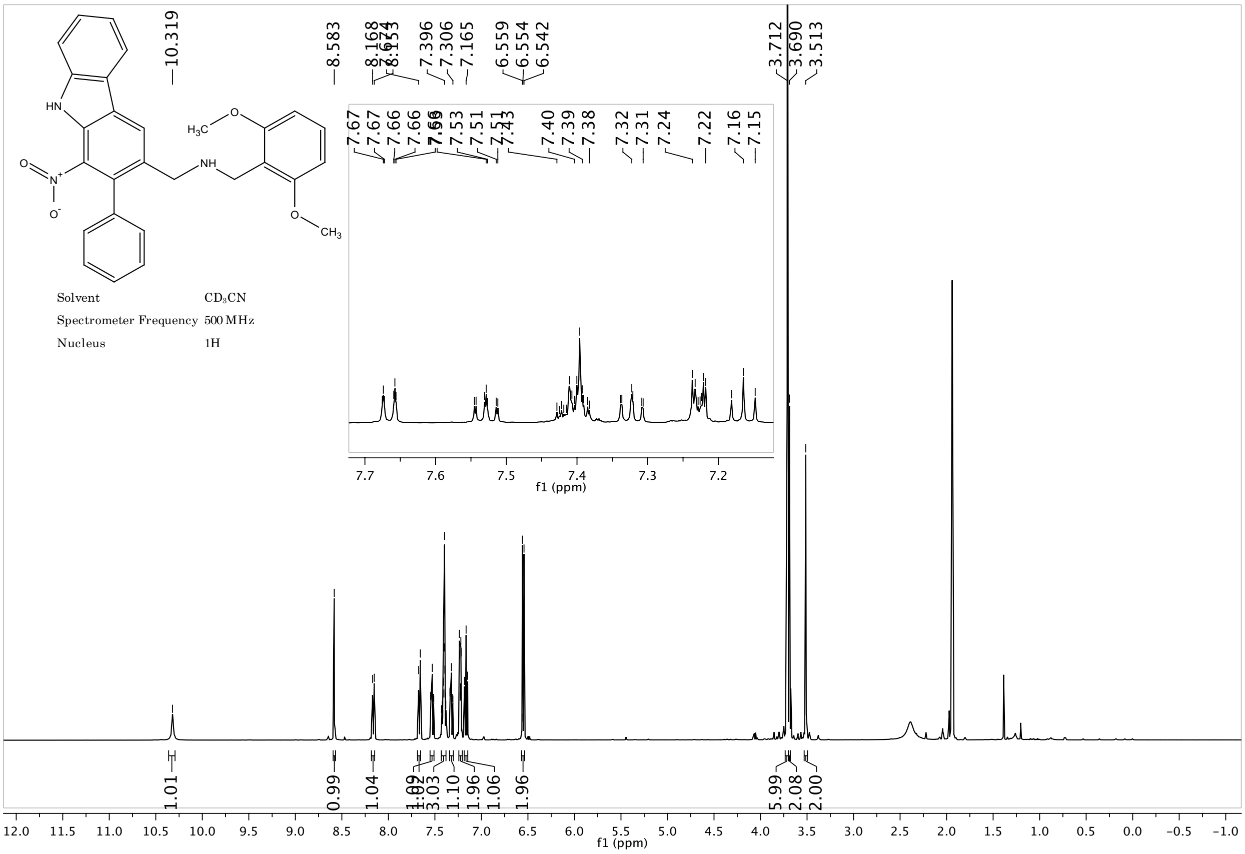

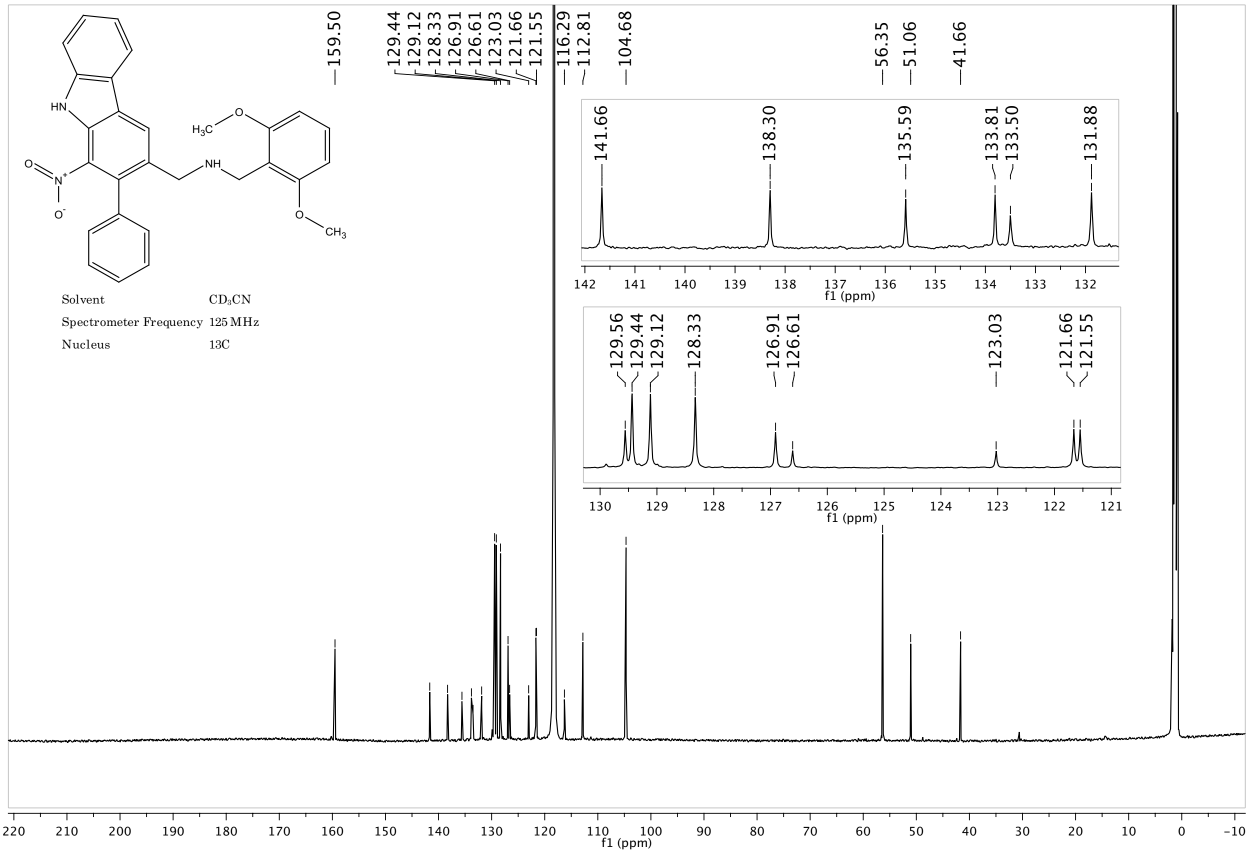

2-(2,6-Dimethylbenzyl)-5-nitro-4-phenyl-3,6-dihydropyrrolo[3,4-c]carbazol-1(2H)-one (15a)

[experimental procedure] [table of contents]

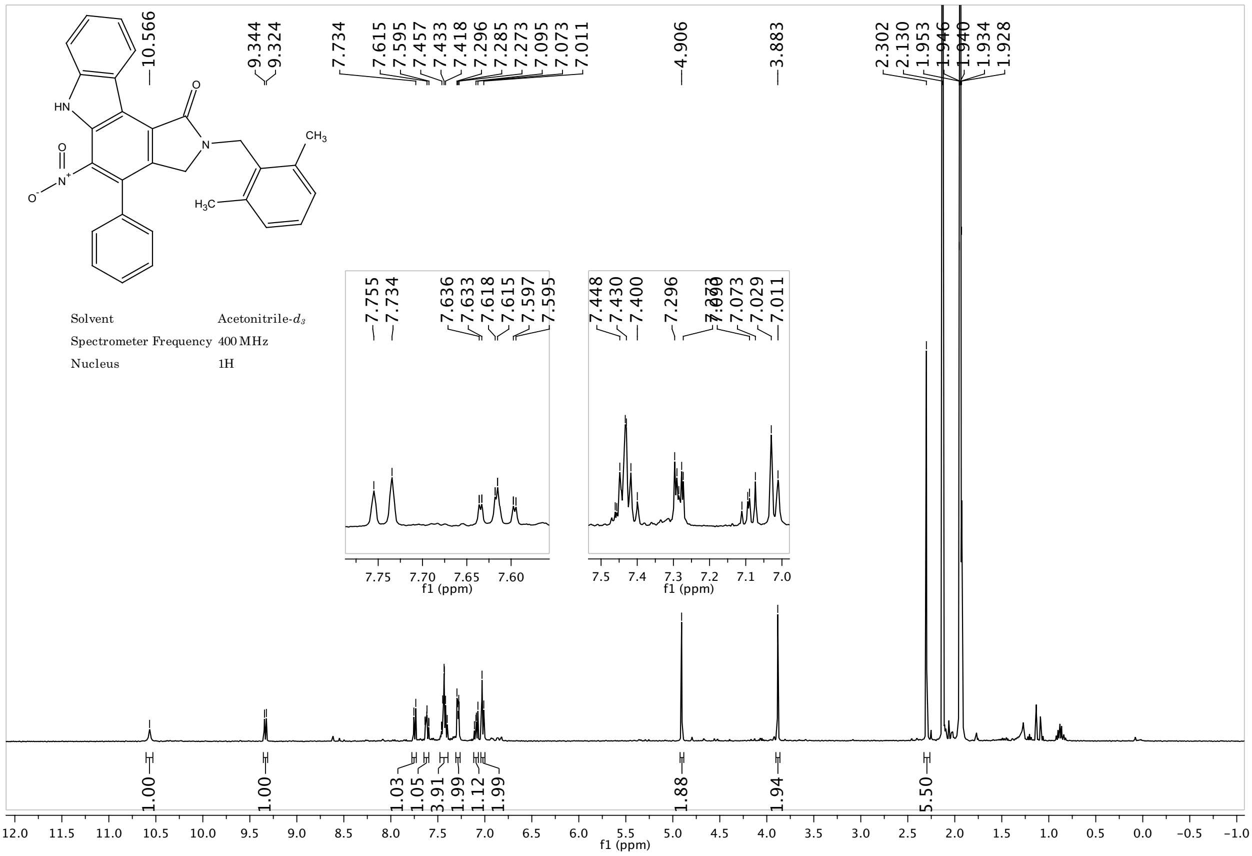

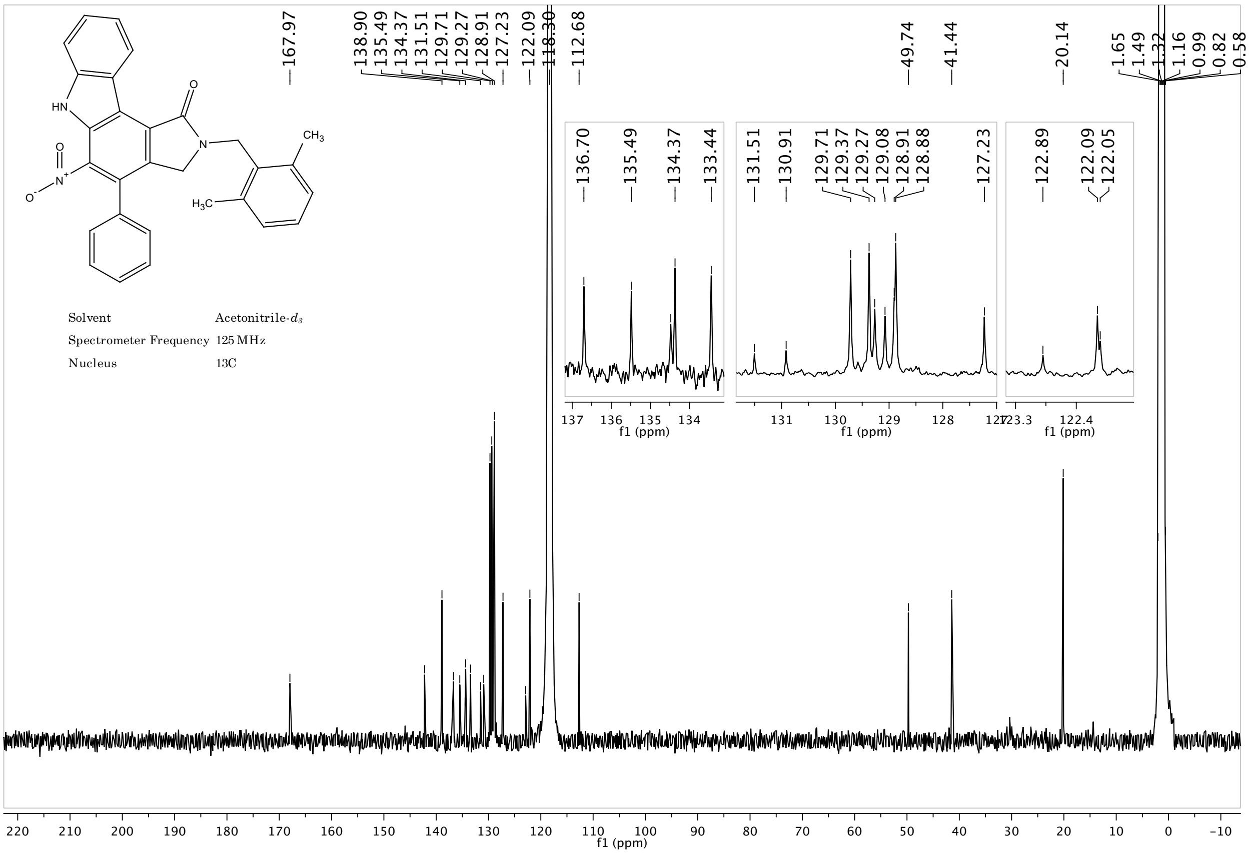

5-Nitro-4-phenyl-2-(2,4,6-trimethylbenzyl)-3,6-dihydropyrrolo[3,4-c]carbazol-1(*2H*)-one (15b)

[experimental procedure] [table of contents]

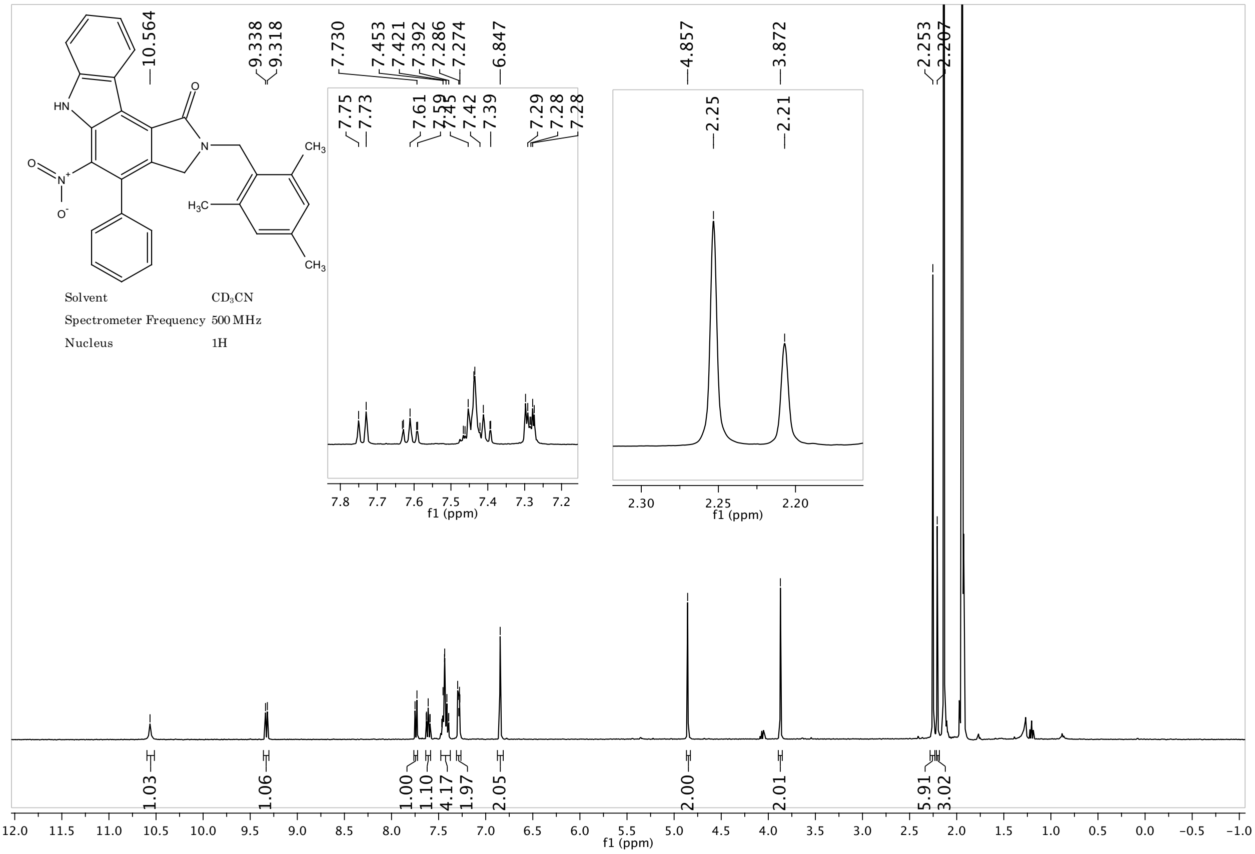

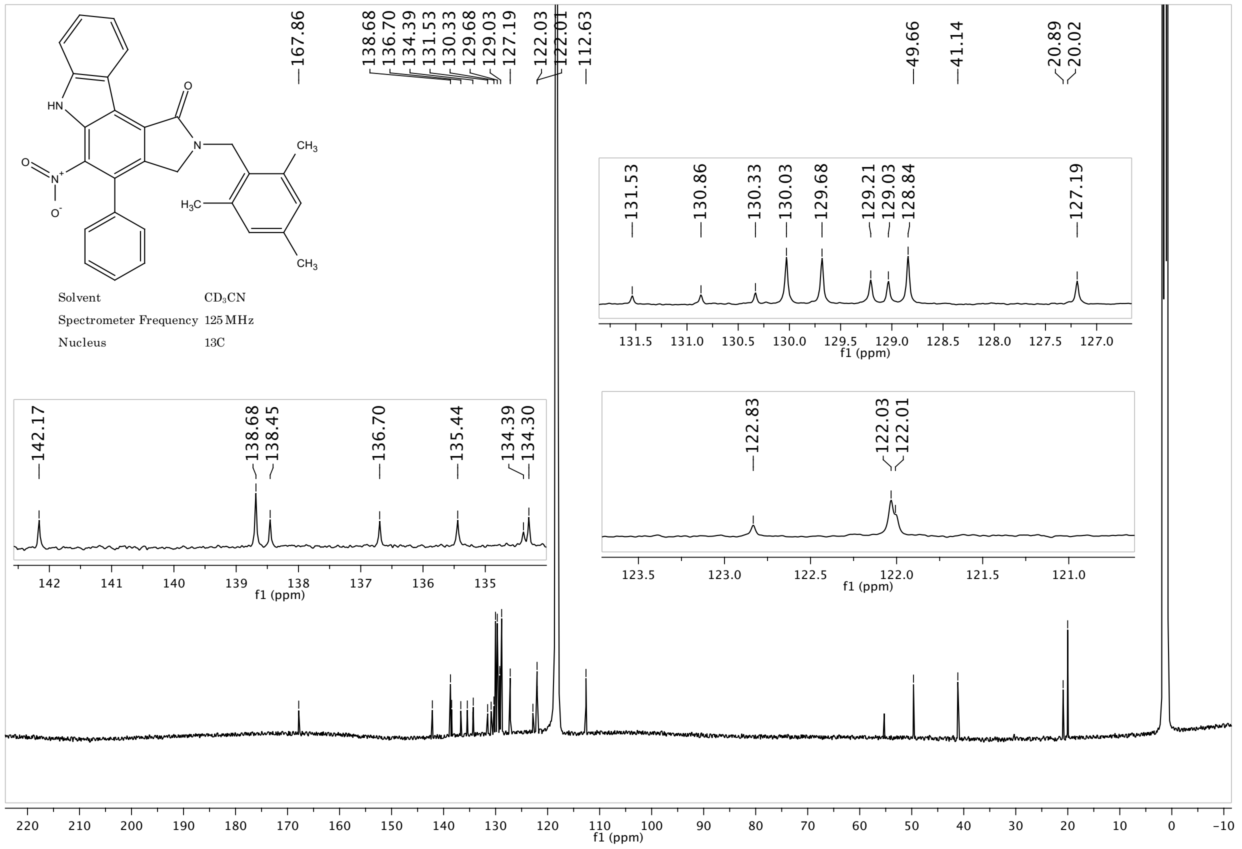

2-(2,6-Dimethoxybenzyl)-5-nitro-4-phenyl-3,6-dihydropyrrolo[3,4-c]carbazol-1(*2H*)-one (15c)

[experimental procedure] [table of contents]

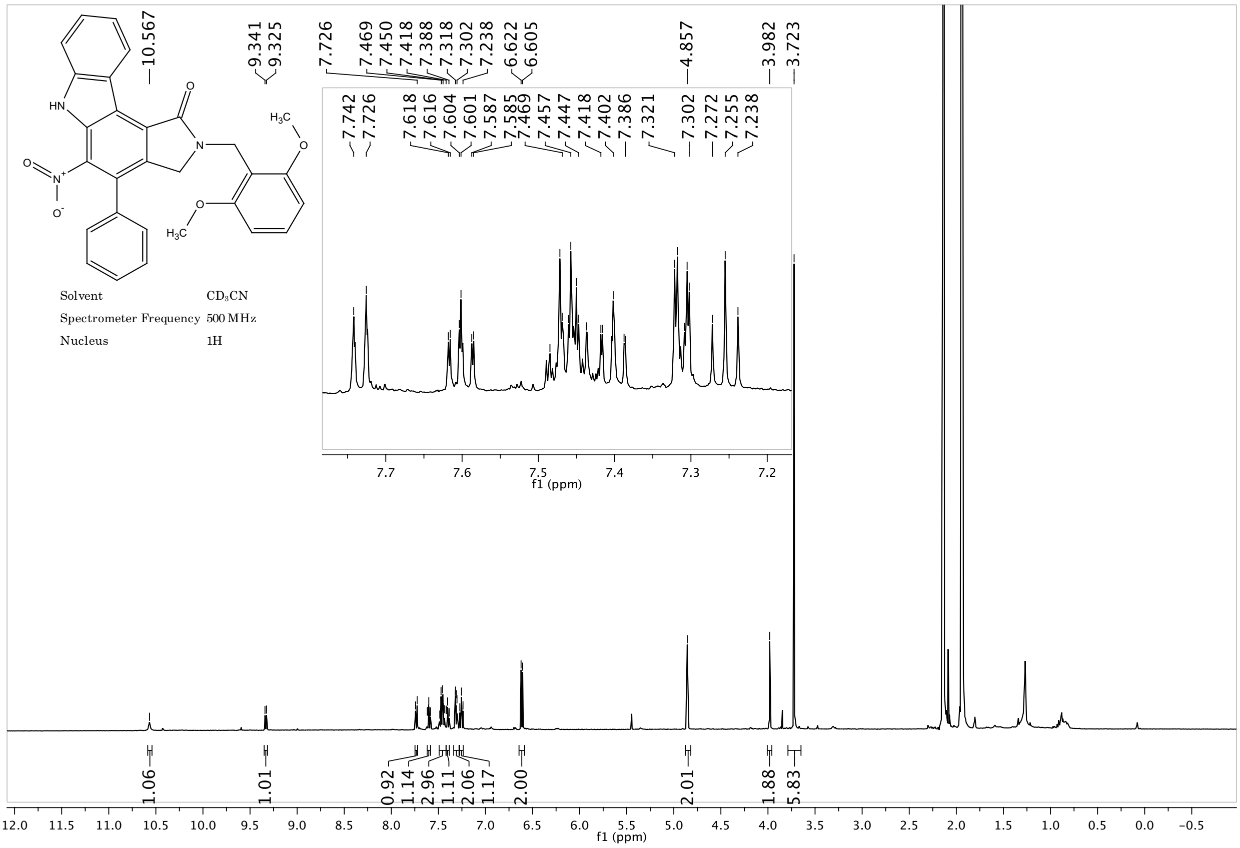

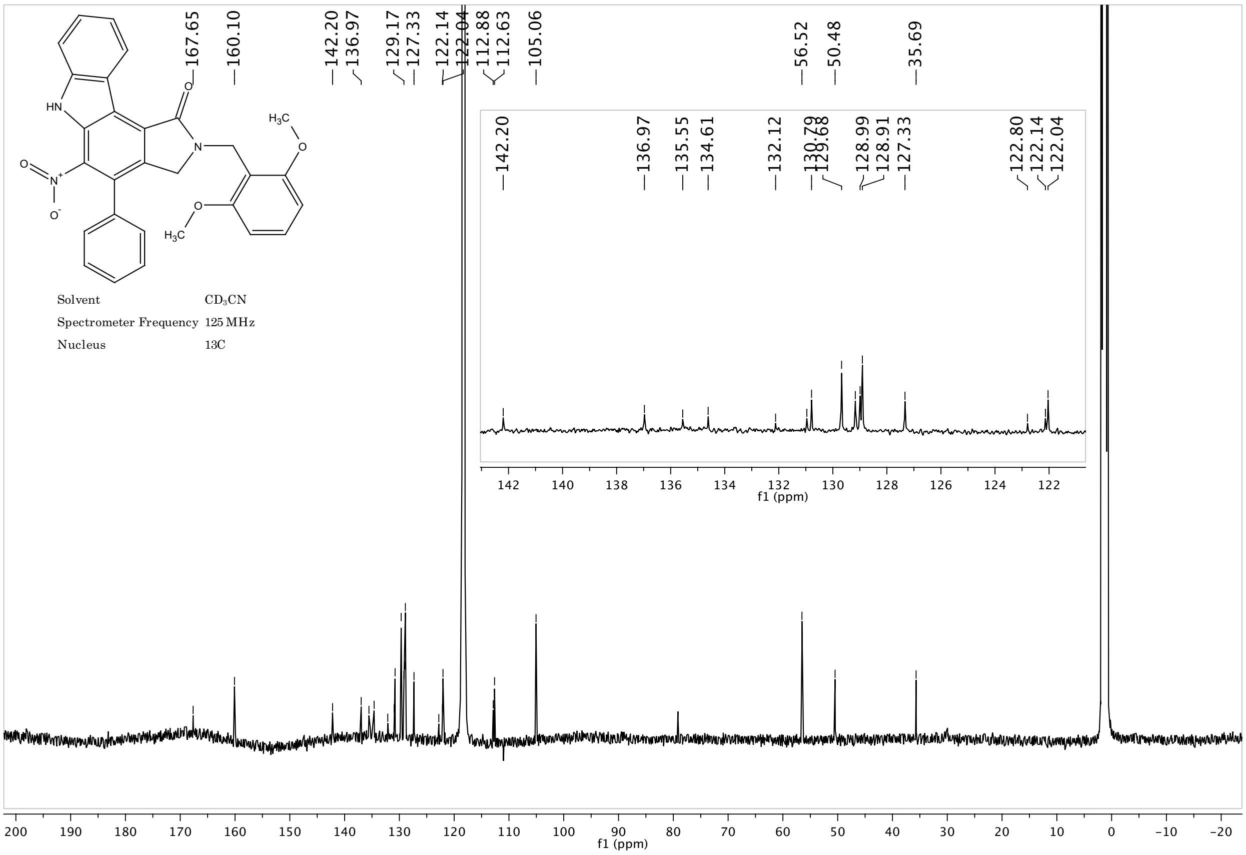

6-(2,6-Dimethylbenzyl)-6,7,12,13-tetrahydro-5*H*-indolo[2,3-a]pyrrolo[3,4-c]carbazol-5-one (16)

[experimental procedure] [table of contents]

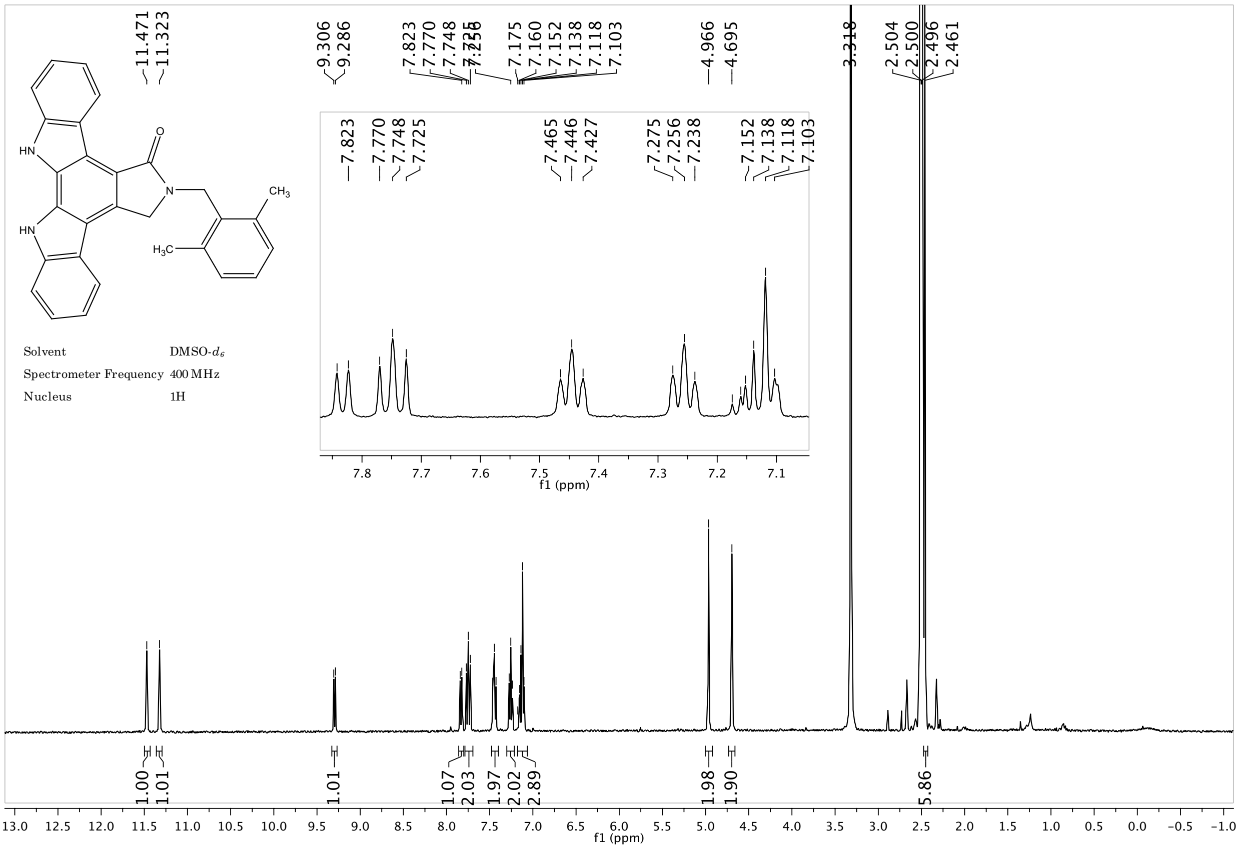

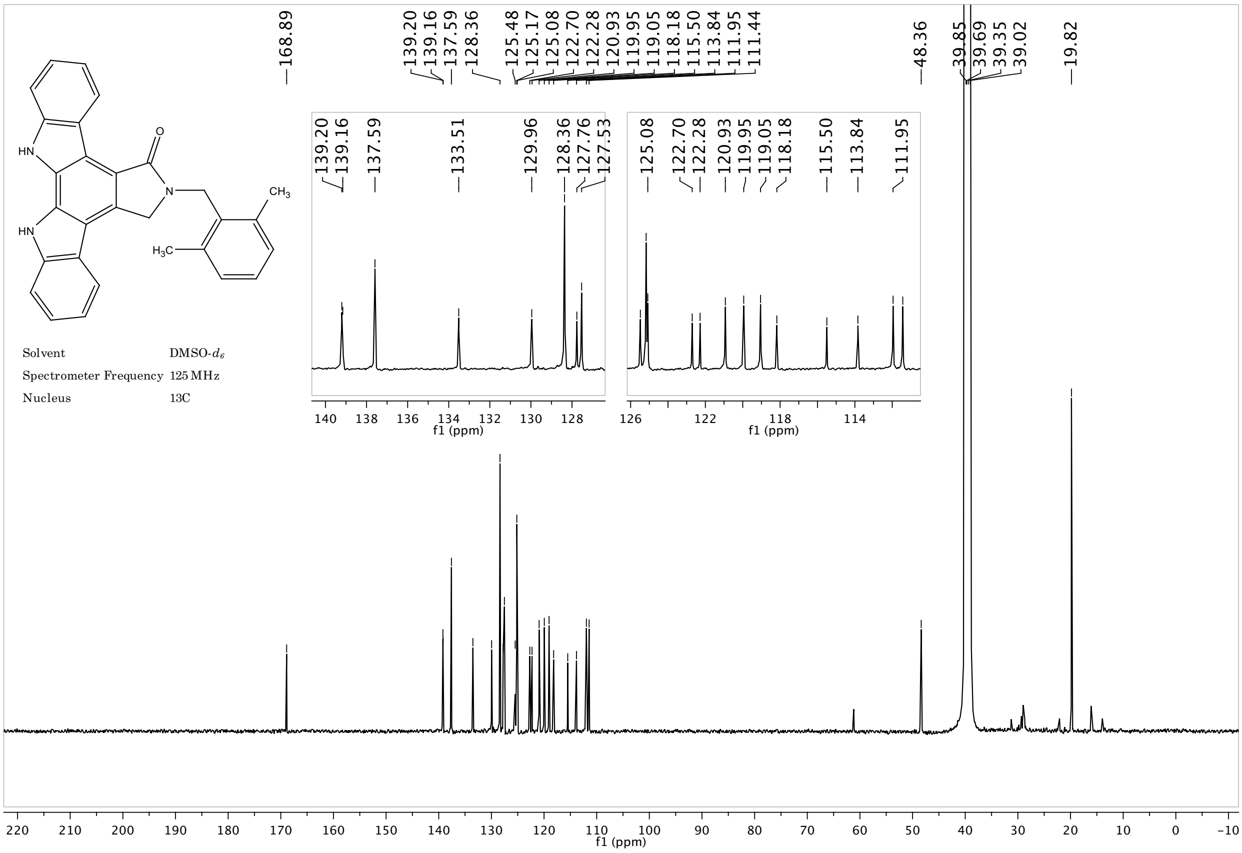

***Staurosporinone* (3)**

[experimental procedure] [table of contents]

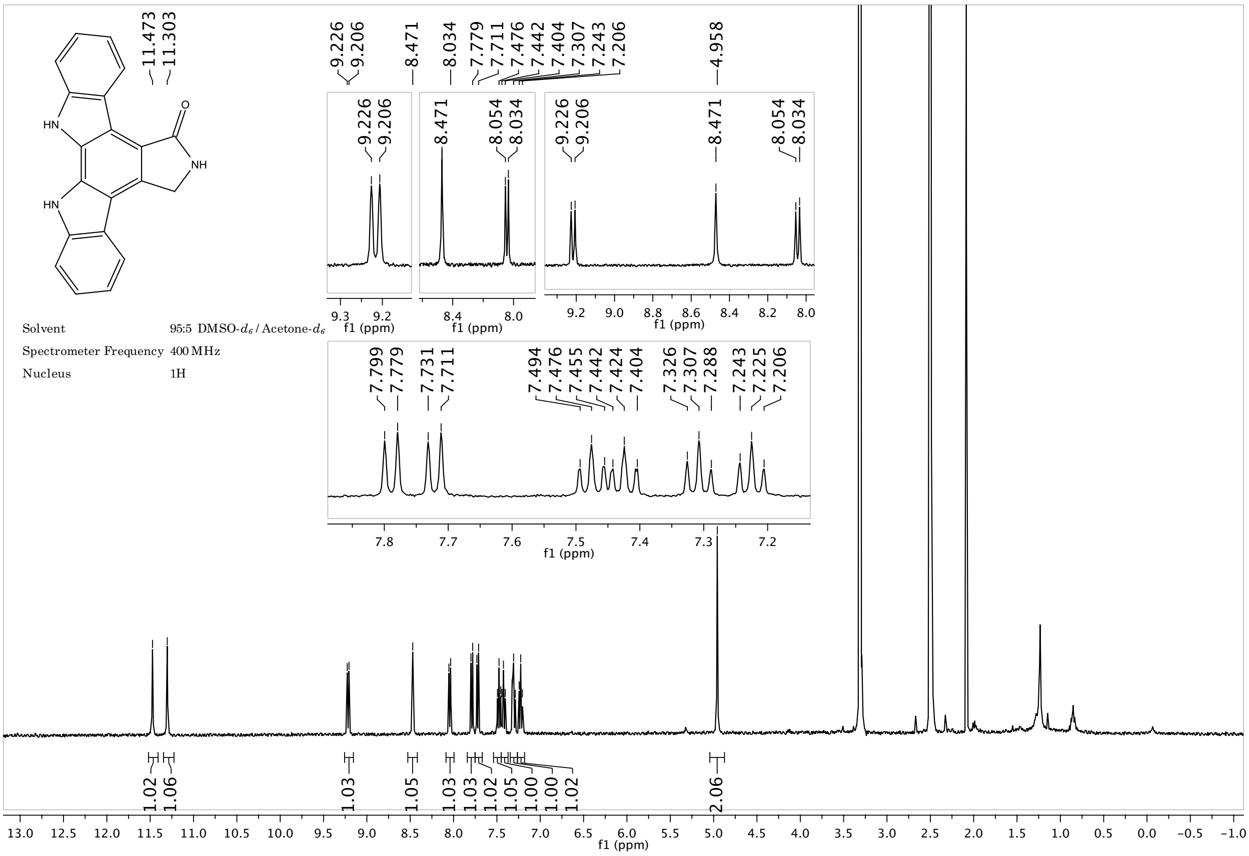

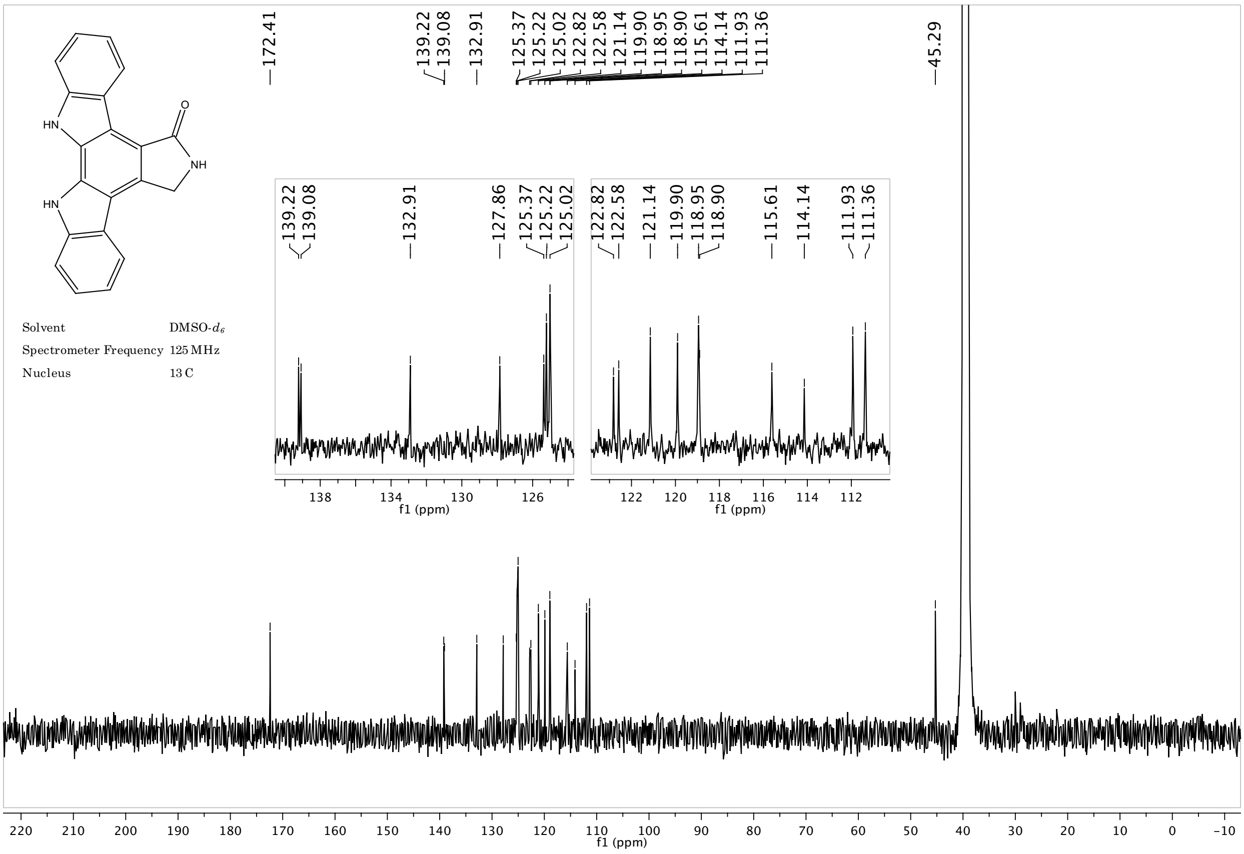

Supplement: Supplementary file 1 [file SC-007-C5SC04399A-s001.pdf]
